# Exploring the potential for an evolutionarily conserved role of the taste 1 receptor gene family in gut sensing mechanisms of fish

**DOI:** 10.1016/j.aninu.2022.08.010

**Published:** 2022-08-31

**Authors:** Anna Rita Angotzi, Esther Leal, Sara Puchol, José M. Cerdá-Reverter, Sofia Morais

**Affiliations:** a Department of Fish Physiology and Biotechnology, Instituto de Acuicultura de Torre de la Sal, IATS-CSIC, Torre la Sal s/n, Ribera de Cabanes, 12595 Castellon, Spain; b Lucta S.A., Innovation Division, Animal Science Unit, UAB Research Park, 08193 Bellaterra, Spain

**Keywords:** Taste 1 receptor, Fish larvae, Gut nutrient sensing, Gut peptides, G(i) alpha protein subunits 1 and 2

## Abstract

In this study, we investigated the transcriptional spatio-temporal dynamics of the taste 1 receptor (*T1R*) gene family repertoire in seabream (*Sparus aurata* [*sa*]), during larval ontogeny and in adult tissues. In early larval development, *saT1R* expression arises heterochronously, i.e. the extraoral taste-related perception in the gastrointestinal tract (GIT) anticipates first exogenous feeding (at 9 days post hatching [dph]), followed by the buccal/intraoral perception from 14 dph onwards, supporting the hypothesis that the early onset of the molecular machinery underlying *saT1R* expression in the GIT is not induced by food but rather genetically hardwired. During adulthood, we characterized the expression patterns of *saT1R* within specific tissues (*n* = 4) distributed in oropharingeal, GIT and brain regions substantiating their functional versatility as chemosensory signaling players to a variety of biological functions beyond oral taste sensation. Further, we provided for the first time direct evidences in fish for mRNA co-expression of a subset of *saT1R* genes (mostly *saT1R3*, i.e. the common subunit of the heterodimeric T1R complexes for the detection of “sweet” and “umami” substances), with the selected gut peptides ghrelin (*ghr*), cholecystokinin (*cck*), hormone peptide yy (*pyy*) and proglucagon (*pg*). Each peptide defines the enteroendocrine cells (ECCs) identity, and establishes on morphological basis, a direct link for T1R chemosensing in the regulation of fish digestive processes. Finally, we analyzed the spatial gene expression patterns of 2 taste signaling components functionally homologous to the mammalian *G(i)α* subunit gustducin, namely *saG(i)α1* and *saG(i)α2*, and demonstrated their co-localization with the *saT1R3* in EECs, thus validating their direct involvement in taste-like transduction mechanisms of the fish GIT. In conclusion, data provide new insights in the evolutionary conservation of gut sensing in fish suggesting a conserved role for nutrient sensors modulating entero-endocrine secretion.

## Graphical abstract

**Figure:**
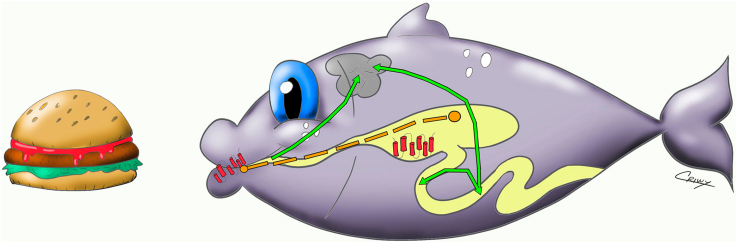

## 1 Introduction

Vertebrates recognize a wide variety of food-related substances by olfactory and taste chemosensory systems to detect chemical cues mediating both appetitive and aversive behaviors to foods. In the classical view, the sense of taste is associated to gustation produced in the oral cavity epithelium, where taste qualities are perceived by specific receptors. In the case of sweet, umami and bitter, taste signaling is initiated by specialized taste G-protein*-*coupled receptors (GPCR) type 1 (T1R) and 2 (T2R), mainly expressed in lingual taste buds (Lindemann, 2001; Chandrashekar et al., 2006). Particularly, the T1R-mediated chemosensing associated to metabolic and hedonic signals initiates in specialized type II-taste receptor cells (TRCs-II) expressing 3 *T1R* gene paralogs (*T1R1*, *T1R2* and *T1R3*) that function as heterodimeric complexes prototypically encoding for sweet (*T1R2*/*T1R3*) or umami (*T1R1*/*T1R3*) taste modalities (Li et al., 2002; Nelson et al., 2002; Finger, 2005). T1R signal transduction within TRCs-II is accomplished via the heterotrimeric G-protein complex G-αβγ that dissociates in the 2 functional components Gα- and Gβγ upon receptor/ligand binding. The best described cellular pathway implicated in mammalian taste transduction relies on the Giα subunit gustducin (Gα-gust)-dependent activation of multiple downstream effectors including phospholipase Cβ2 (PLCβ2), inositol triphosphate receptor 3 (IP_3_R3) and transient receptor ion channel 5 (TRPM5), ultimately leading to the elevation of intracellular calcium, taste cell membrane depolarization and afferent neuronal transmission to the gustatory cortex (reviewed by Ahmad and Dalziel, 2020).

A multitude of studies in the last decades uncovered that the role of T1R and T2R in chemosensing is not limited to canonical gustatory functions driving food choices towards ingestion or rejection, but it rather extends far beyond oral cavity sensing (reviewed by Finger and Kinnamon, 2011). Indeed, T1R and T2R expression and associated signaling pathways have been identified in extra-oral tissues of endodermic origin (i.e. digestive and respiratory apparatuses), within a large polymorphic population of isolated or clustered cells presumably involved in immune and digestive functions, and collectively recognized as the diffuse chemosensory system (DCS) (Braun et al., 2011; Uhlen et al., 2015; Hass et al., 2010; Taniguchi, 2004; Sbarbati and Osculati, 2003, 2005). Accordingly, expression of T1R has been documented in mammalian enteroendocrine cells (EECs) of the gastrointestinal tract (GIT), along with functional evidences on their implication in the modulation of gut hormone release. Gut peptides secreted upon T1R activation in the GIT are important endocrine factors responsible for the regulation of many physiological processes including satiation and satiety, digestive (acid, bile and enzyme secretion, and gut motility) and absorptive (nutrient transporter expression and nutrient uptake) functions, epithelial cell proliferation and regeneration as well as metabolism (energy and glucose homeostasis) (Dyer et al., 2005; Depoortere, 2014; Raka et al., 2019; Jang et al., 2007; Alpers, 2010).

From an evolutionary perspective, the 3 *T1R* orthologs are conserved across vertebrates, including fish, whose *T1R* families have greatly expanded mostly due to additional *T1R2* duplicates that apparently evolved to increase taste plasticity for amino acid sensing (Hashiguchi et al., 2007; Baldwin and Ko, 2020; Oike et al., 2007; Angotzi et al., 2020). Emerging evidences based on quantitative molecular studies indicate that several taste receptors and canonical components of T1R transduction signaling are also present in the fish GIT, suggesting that the T1R-mediated gut sensing mechanisms could have been conserved during evolution (Polakof and Soengas, 2013; Latorre et al., 2013; Ronnestad et al., 2016; Calo et al., 2021). On the other hand, the Gα-gust system is absent in the genome of both amphibians and teleost fishes as a result of 2 independent gene losses in their last common ancestors (Oka and Korsching, 2011; Ohmoto et al., 2011), somehow implying that other G(i)α-related proteins might be involved in the initial steps of taste signaling in these lineages. In line with this hypothesis, in a recent study where we comprehensively described the *T1R* gene repertoire of the carnivorous marine fish gilthead seabream (*Sparus aurata* [*sa*]), and it was also shown in vitro that heterologous expression of *saT1R* heterodimers co-transfected with the Gi alpha protein subunits saG(i)α1 and saG(i)α2 triggered both stimulatory and inhibitory taste transduction mechanisms upon amino acid activation (Angotzi et al., 2020). Hence, the overall emerging picture suggests a large degree of conservation of the T1R-mediated taste signaling across vertebrates, including fish.

However, despite the important progresses made to describe the functional and evolutive aspects of T1R and related taste signaling cascades in teleosts, many basic aspects of T1R biology remain largely unexplored. For instance, a putative element of the taste signaling pathway, namely Gi alpha protein-like immunoreactivity, has been localized in the GIT, in cells with an endocrine appearance, co-localizing with some peptides in the fish stomach (Latorre et al., 2013), but to our knowledge there is no direct evidence linking the presence of T1R and gut hormones in the same cell type (specifically EECs). Such evidence would be a fundamental stepping stone towards establishing the possible existence of gut sensing mechanisms operating in fish similarly to mammals. To the best of our knowledge, no study has characterized the ontogeny of the *T1R* gene system in early life stages of fish. Indeed, only a few published studies examined developmental aspects related to the fish gustatory system mostly focused on taste bud morphology (Hansen et al., 2002; Wang et al., 2016), cell patterning and distribution (Varatharasan et al., 2009) or development of oral taste functionality by behavioral methods (Kasumyan, 2001).

Having in mind these knowledge gaps, the objective of this study was to address aspects related to spatio-temporal gene expression patterns, and obtain anatomical information on the full set of *saT1R* genes in fish larvae at different stages of ontogeny and in selected tissues during adulthood. Moreover, we analyzed the specific gene expression patterns of the 2 signal-transducing components *sa*G(i)α1 and *sa*G(i)α2 to establish their potential co-localization with the *saT1R3* gene expression, as the common subunit of T1R heterodimeric complexes, in the GIT of adult fish. Finally, we aimed to provide direct evidence for mRNAs co-expression of a subset of *saT1R* genes (mostly *saT1R3*) with selected gut peptides defining EEC-type identity such as ghrelin (*ghr*), cholecystokinin (*cck*), peptide YY (*pyy*) and proglucagon (*pg*), to establish a morphological link indicating possible roles of T1R chemosensing in gut nutrient-sensing mechanisms and in the regulation of fish digestive processes.

## 2 Materials and methods

### 2.1 Animals and ethical statement

Gilthead seabream adults and newly hatched larvae were obtained in January 2018 from the fish farm Avramar (Spain), and were maintained in fiber-glass aerated tanks supplied with a continuous flow of seawater (37 g/L salinity, 16.9–17.2 °C), and under a natural photoperiod at the facilities of the IATS institute (CSIC, Torre la Sal, Spain). Following the complete absorption of the yolk sac at 8 d post hatching (dph), larvae were fed on rotifers once per day from 9 to 17 dph, and gradually replaced by a mixed diet of rotifers and *Artemia* naupli as development progressed, until the last day of sampling (21 dph). Adult fish were fed twice daily on a standard commercial diet (Biomar, Spain), and were fasted for 24 h prior to tissue sampling. All experimental procedures were performed in compliance with the European Union guidelines for Care and Use of Laboratory Animals (2010/63/EU), and after the approval of the Welfare and Bioethical Committee of Instituto de Acuicultura de Torre de la Sal (IATS-CSIC) under the code 015/2013 and according to Royal Decree RD53/2013.

### 2.2 Quantification of *saT1R* mRNA abundance by real-time quantitative PCR (RT qPCR)

Real-time quantitative PCR analyses of *saT1R1*, *saT1R2a*, *saT1R2b*, *saT1R2d*, *saT1R2e*, *saT1R2f* and *saT1R3* genes were performed using RNA pools of whole seabream larvae collected at 1, 3, 5, 7, 10 and 12 dph, i.e. spanning life–stage transition from yolk-sac sustenance to exogenous feeding (initiated at 9 dph). For each stage analyzed, total RNA was extracted from triplicate samples, each containing approximately 15 pooled whole-body larvae. For qPCR analyses of adult tissues, 4 fish (*n* = 4; 348 ± 53 g) were euthanized with an overdose of tricaine methane-sulfonate (MS-222; 400 mg/L), and tissue samples ranging from 50 to 100 mg were dissected from the oropharyngeal area, including lips (L), gill filaments (G), the epithelium overlying the bony basyhyal (homologous to the tongue of tetrapods, T), and the mucous epithelium lining the inside of the oral cavity (OC). For GIT tissue sampling, stomach (St; posterior part) and intestine samples were dissected. The intestine was first equally divided into 3 major antero-posterior segments; then the middle portion of each segment was dissected for further processing and hereby defined as foregut (Fg), midgut (Mg) and hindgut (Hg). The 3 brain tissue compartments analyzed included telencephalic/hypothalamic (Forebrain, Fb), mesencephalic (midbrain, Mb) and romboencephalic (hindbrain, Hb) regions, respectively. Larvae and dissected tissues were mechanically homogenized in 1 mL TRIzol reagent (Invitrogen, St. Louis, MI, USA), and the concentration and purity of RNAs were determined by the optical density 260/280 ratio (>1.9), using a NanoDrop 2000c spectrophotometer (Thermo Scientific, United States). To eliminate potential genomic DNA, samples were treated with the TURBO DNA-free kit (Ambion, Life-Technologies, Austin, TX, USA) according to the supplier's protocol. The cDNAs were synthesized from 2 μg of DNase-treated RNAs using oligo(dT)12-18 primer and Superscript III (Invitrogen, Carlsbad, CA, USA) following the manufacturer's instructions. Target mRNAs were quantified in duplicate samples by real-time qPCR (Bio-Rad CFX96) using Sybr green PCR master mix (Invitrogen), 300 nM of forward and reverse primers, 50 ng cDNA template and nuclease-free water up to a final volume of 25 μL. All primers were designed using the free software OligoAnalyzer Tool (Integrated DNA technologies), to ensure similar melting temperatures, avoidance of self and hetero dimerization and a balanced G/C content. Sequences of primers used for reference and target genes are listed in Supplementary Table S1. Primer pair efficiency (E) was evaluated using a 2-fold dilution curve ranging from 100 to 6 ng cDNA pools, and was determined by formula E (%) = (10^−1/slope^ − 1) × 100; the primers with efficiency in the range of 95% to 105% were selected for quantitative gene expression analysis. PCR conditions were as follow: 50 °C for 2 min; 95 °C for 10 min; 40 cycles of 95 °C for 15 s, 60 °C for 1 min. Melting curve analysis to evaluate potential non-specific amplification was performed by ramping from 60 to 92 °C, rising by 0.2 °C every 1 s. Specificity of amplified PCR products was further confirmed by electrophoresis on a 1.2% agarose gel. Fold-change gene expressions of target transcripts were estimated using the mean normalized expression method of the Q-Gene application (Muller et al., 2002; Simon, 2003), using the stably expressed gene-elongation factor 2 as internal reference for data normalization. Relative gene expression results are expressed as the mean ± SEM. Statistical comparisons were analyzed by one-way analysis of variance (ANOVA) followed by Tukey multiple test, using GraphPad Prism 8. A *P*-value < 0.05 was considered to be statistically significant.

### 2.3 Whole-mount mRNA in situ hybridization (WISH)

To enable visualization of *saT1R*'s transcripts using WISH, larvae of 5, 11, 14, 17 and 21 dph were fixed in 4% paraformaldehyde (PFA; Sigma-Aldrich, Gillingham, UK) in phosphate-buffered saline (PBS) solution (pH 7.4) for 48 h at 4 °C. If not mentioned otherwise, all working steps were performed at room temperature. Specimens were then washed in PBS, dehydrated through a graded methanol series and preserved at −20 °C for long-term storage. WISH procedure was performed as described by Thisse and Thisse (2008). Briefly, larvae were rehydrated in methanol series in PBS containing 0.15% Tween-20. Bleaching of the larvae pigment was accomplished by immersion in 3% H_2_O_2_/0.5% KOH for 30 min. Specimens were permeabilized for 20 min with 10 μL/mL of proteinase K (Promega) in 0.05 M Tris-HCl (pH 7.5) for 11 to 14 min, depending on stage. Hybridization with digoxigenin (DIG) antisense riboprobes (700 ng/mL of hybridization buffer (HB): 50% deionized formamide; 300 mM NaCl; 10 mM Tris-HCl (pH 7.5); 1 mM EDTA (pH 8); 1% blocking reagent (Sigma); 10% dextran sulfate (Sigma); 0.2% Tween20), was performed by overnight incubation (O/N) at 65 °C, followed by washing steps at 65 °C in 50% formamide in 2XSSC (2 × 5 min, 1 × 20 min, 1 × 30 min), in 2XSSC containing 0.15% Tween-20 (2XSSCT; 3 × 15 min) and in 0.2XSSCT (2 × 25 min). Embryos were treated with 20 μg/mL RNase A in RNase Buffer (50 mM NaCl, 10 mM Tris-HCl, 5 mM EDTA) for 30 min at 37 °C, and washed in Immuno buffer (2XSSC, 2% blocking reagent, 0.05 Triton x-100) for 2 h, followed by O/N incubation at 4 °C with anti*-*DIG antibodies conjugated to alkaline phosphatase (AP) diluted 1:2,000 in antibody solution containing 1 × maleate buffer (100 mM maleic acid, 150 mM NaCl, pH 7.5) in 1% blocking reagent and 0.05% Triton x-100 (Sigma). mRNA signals were visualized with 75 mg/mL of 4-nitro blue tetrazolium chloride (NBT) and 50 mg/mL of 5-bromo-4-chloro-3-indolyl-phosphate (BCIP) (Roche Diagnostics, Germany) in buffer 2 (100 mM Tris-HCl pH 9.5, 5 mM MgCl_2_, 100 mM NaCl), containing 1 mM levamisol. To stop the staining reaction, larvae were subsequently washed in buffer 3 (10 mM Tris-HCl (1 mM EDTA pH 8.0, 150 mM NaCl, pH 7.5) (2 × 30 min) and washed 30 min in PBST prior fixation in PFA for 48 h at 4 °C to stabilize mRNA signals. Finally, all samples were washed thoroughly in 1× PBS and stored at 4 °C in 70% glycerol/1 × PBS until visualization with a SZX16 stereomicroscope equipped with an SDF PLAPO 2×PFC objective (Olympus, Japan). Control experiments using proopiomelanocortin β (*pomcβ*) antisense probes and selected *saT1R* sense probes were run in parallel as positive controls of the WISH assay or to detect potential non-specific *T1R* signals, respectively.

### 2.4 Chromogenic (CISH) and dual fluorescent mRNA in situ hybridization (FISH)

To prepare specimens for mRNA in situ hybridization on tissue sections, samples from stomach, pyloric caeca, proximal, middle and distal intestines were all dissected and immediately perfused with ice-cold PBS until the effluent was clear, fixed in PFA for 48 h at 4 °C, and then dehydrated through a graded ethanol series before being embedded in paraffin wax. Sections of 7 μm were prepared on a Microm HM 355 microtome (Fisher scientific), collected on poly-L-lysine coated slides (VWR, Germany) and preserved at −80 °C until the experiments were performed. Prior to in situ hybridization (ISH) procedures, slides were air-dried for 1 h and heated at 60 °C for 10 min. Deparaffinization and rehydration were performed in xylene and ethanol series, 100% xylene (3 × 5 min), 100% ethanol (2 × 3 min), 95% ethanol (1 × 3 min), 70% ethanol (1 × 3min) and 50% ethanol (1 × 3min). Tissue sections were then fixed in 4% PFA/PBS (pH 7.4) (1 × 10 min), and permeabilized in 10 μg/mL of Proteinase K in 0.05 M Tris-HCl (pH 7.5; 1 × 10 min) followed by washes in 1× PBS (1 × 5 min) prior additional fixation in 4% PFA/PBS (1 × 10 min). Sections were rinsed in 1× PBS (2 × 5 min) and treated with freshly made acetylation solution containing 0.25% acetic anhydride and 0.2% HCl in 0.1 M triethanolamine buffer (pH 8; 1 × 10 min). After rinsing again in 1 × PBS (3 × 5 min), tissues were rehydrated in a graded series of ethanol (95, 80 and 70%, 1 min each) and pre-hybridized in HB for 90 min; HB was then removed and replaced by one (for single FISH or CISH) or 2 (for dual FISH) cRNA probes. Different cRNA concentrations between 1 and 20 ng/μL were tested, and the best results were achieved using 8 ng/μL. Sections were covered with Grace Bio-Labs HybriSlip (Sigma Aldrich) and incubated for 16 h at 63 °C in humidified chamber with wipes soaked in 5XSSC. Post-hybridization washes included 2XSSC (1 × 30 min) and 50% formamide in 2XSSC (1 × 30 min) at 63 °C, followed by 10 μg/mL RNase A treatment, for 30 min at 37 °C.

For CISH procedure, slides were incubated in Immuno buffer containing 1:2,000 diluted anti-DIG/Fab fragments conjugated to AP and incubated O/N at 4 °C. To develop the staining, sections were then washed in buffer 2 and incubated O/N at 4 °C with NBT/BCIP chromogen substrates. Finally, sections were washed in buffer 3 (1 × 30 min) to stop the reactions and mounted on 60% glycerol in buffer 3 until visualization.

For dual FISH detection, experiments were carried out as described by Hoang et al. (2016). Briefly, subsequent to the RNase A treatment, sections were incubated O/N at 4 °C with an antibody solution containing 1:250 diluted anti-fluorescein Fab fragments conjugated with horse-radishperoxidase antibody (Sigma Aldrich). After several washes in 1× maleate buffer (2 × 10 min) and TNT buffer (100 mM Tris-HCl pH 7.5, 150 mM NaCl, 0.05% Tween-20), (3 × 5 min), green fluorescent signals were developed by tyramide stock solution 1:200 using 1× Plus Amplification Diluent, according to the manufacturer's specifications (TSA PLUS fluorescein kit, PerkinElmer). The reaction was subsequently stopped in buffer 3 (4 × 5 min), and the slides were incubated O/N in humidified chamber at 4 °C with antibody solution containing 1:2,000 diluted anti-DIG/Fab fragments conjugated to AP, as previously described for single CISH detection. Sections were then washed in visualization buffer (100 mM Tris-HCl pH 8.2 and 0.1% Tween-20) and red fluorescent staining visualized with SIGMA FAST TR/naphtholAS-MX/Tris buffer, prepared according to the manufacturer's procedures (Sigma, Aldrich). For blue fluorescent DNA stain, slides were incubated (1 × 1 min) in 0.5 μM of bisBenzimide H 33342 trihydrochloride (Hoechst; Invitrogen). Finally, cover slips were mounted onto slides using Pro-Long Gold mounting media (Thermo Fisher scientific). Images were visualized with BX41 fluorescent microscope (Olympus), using 10× and 20× objectives, and documented with cellSens 1.18 software (Olympus), followed by brightness and contrast adjustments using Adobe Photoshop CS5 (Adobe Systems Inc., San Jose, CA), before being analyzed and mounted on multi-panel figures.

### 2.5 Riboprobes preparation

The DIG and fluorescein-labeled riboprobes for *sa*G(i)α1-2, *saT1R1*, neurogenic differentiation 1 (*nd1)*, *ghr*, *cck*, *pyy*, *pg* and *pomcβ* genes were all synthesized from PCR-amplified DNA fragments using the primers listed in Table S1**.** All PCR-products were cloned into pGEM-T Easy vector plasmids (Promega, Madison, WI), and were Sanger-sequenced to confirm DNA specificity. Linearized gene specific plasmids were then subjected to in vitro transcription using 25 U of SP6 or T7 RNA polymerases (Promega) in the presence of DIG-labeled or Fluorescein-labeled UTPs, following the manufacturer's instructions (Roche Diagnostic). Synthesized cRNA probes were subsequently precipitated with 2.5 × 100% ethanol/LiCl (3M), and spectrophotometrically quantified. To make sure that the different cRNA probes used in multiple ISH do not cross-react, we aligned the cRNA sequences of interest using CLUSTALX V1.81. Probe sizes and percentage of nucleotide identity between the conserved sequence targets of the T1R's probes are presented in Table S1 and Supplementary Table 2 (Table S2), respectively.

## 3 Results

### 3.1 *saT1R* qPCR in whole larvae

**Fig. 1:**
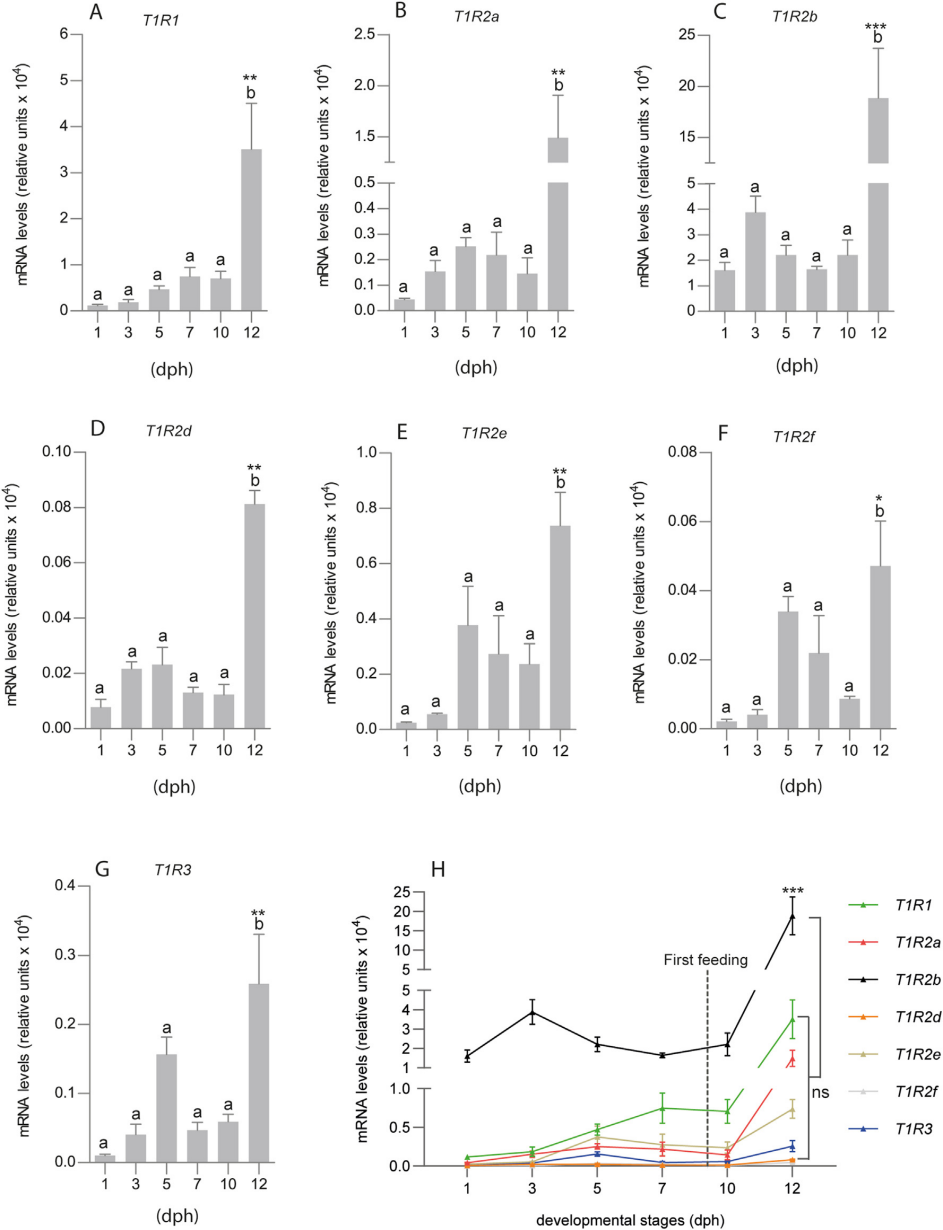
Graphical representation of RT qPCR analyses in whole seabream larvae for seabream (*sa*) taste 1 receptor (*T1R*) subunits, *saT1R1* (A), *saT1R2a* (B), *saT1R2b* (C), *saT1R2d* (D), *saT1R2e* (E), *saT1R2f* (F), *saT1R3* (G) at 1, 3, 5, 7, 10, 12 d post hatching (dph). *saT1R2b* mRNAs expressed roughly 300-fold higher levels of *saT1R2f* and 60, 30, 10 and 5-folds of sa*T1R3*, sa*T1R2e*, sa*T1R2a* and sa*T1R1*, respectively (H). Data are represented as the fold change differences of target gene expression to the reference gene-elongation factor 2, per 100 ng of input RNA/sample. All experiments were performed in triplicates (*n* = 3), each containing approximately 15 pooled whole-body larvae; data are expressed as means ± standard error of the mean (SEM). Different letters indicate significant differences between experimental groups. Asterisks indicate significance levels (*P < 0.05, **P < 0.01, ***P < 0.001) after one-way ANOVA followed by Tukey's multiple comparisons tests (GraphPad Prism version 8.0).

To characterize the ontogeny of *saT1R*'s gene expression, and unveil potential temporal variations in relation to first-feeding, qPCR analyses of each *saT1R* gene were performed using mRNA pools of whole seabream larvae from 1 until 12 dph, i.e. spanning life–stage transition from yolk-sac sustenance to exogenous feeding (initiated at 9 dph). The 7 *saT1R* transcripts were stably expressed with no significant variations (*P* > 0.05) among stages from 1 until 10 dph (Fig. 1A–G). Shortly after the beginning of exogenous feeding, at 12 dph, all *saT1R* significantly increased their expression levels, with *saT1R2b* being the most responsive gene (*P* < 0.001; Fig. 1C), followed by *saT1R1*, *saT1R2a*, *saT1R2d*, *saT1R2e* and *saT1R3* (*P* < 0.01; Fig. 1A, B, D, E, G); the least significant increases were observed for the *sa*T1R2f transcript (*P* < 0.05; Fig. 1F). Statistical comparisons of *saT1R* mRNA expression levels at 12 dph, indicated that *saT1R2b* was expressed roughly 300-fold higher than *saT1R2f*, and 60, 30, 10 and 5-fold higher than *saT1R3*, *saT1R2e*, *saT1R2a* and *saT1R1*, respectively (*P* < 0.001 for all comparisons). No significant differences were observed among *saT1R1*, *saT1R2a*, *saT1R2d*, and *saT1R3* transcripts at the same stage (Fig. 1H).

### 3.2 *saT1R* qPCR in adult tissues

**Fig. 2:**
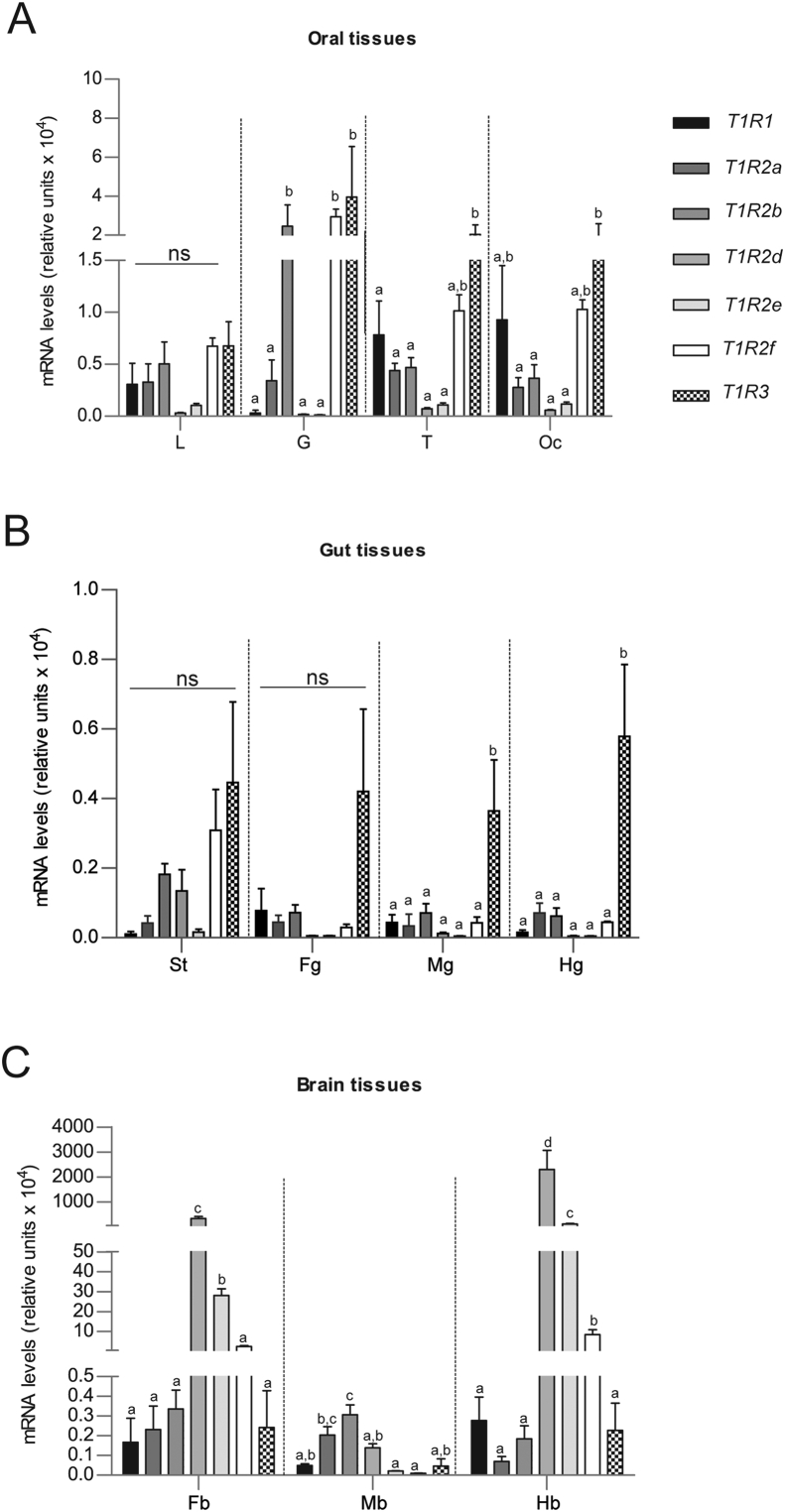
Graphical representation of RT qPCR analyses of seabream (*sa*) taste 1 receptor (T1R) subunits, *saT1R1*, *saT1R2a-f* and *saT1R3* in adult seabream tissues of oropharyngeal (A), gastrointestinal tract (B) and brain tissues (C). Data are represented as the fold change differences of target gene expression to the reference gene-elongation factor 2, per 100 ng of input RNA/tissue. L = lips; G = gill filaments; T = tongue; Oc = oral cavity epithelium; FG = foregut; MG = midgut; HG = hindgut; FB = forebrain; MB = midbrain; HB = hindbrain. All experiments were performed in quadruplicates (*n* = 4); data are expressed as means ± standard error of the mean (SEM). Different letters indicate significant differences after one-way ANOVA followed by Tukey's multiple comparisons tests (see section of results for significance levels).

Expression profiles of the 7 *saT1R* genes were also examined by qPCR in several adult tissues. Their distribution patterns were visualized by plotting the relative mRNA abundance of each *saT1R* gene in 3 regions: oropharyngeal, GIT and brain tissues. In the oropharyngeal region (Fig. 2A), the 7 *saT1R* gene transcripts were found in all tissues investigated (lips, tongue, gill filaments and oral cavity epithelium), and significantly higher expression levels were found for the following: 1) *saT1R3* in gills (*P* < 0.05 for *saT1R1*/*saT1R2a*/*d*/*e* comparisons), tongue (*P* < 0.05 and *P* < 0.01, for *saT1R1* and *saT1R2a*/*b*/*d*/*e* comparisons, respectively) and in the oral cavity epithelium (*P* < 0.05, for *saT1R2a*/*b*/*d*/*e* comparisons), and 2) *saT1R2b* and *saT1R2f* genes in the gills (*P* < 0.01 for *saT1R1*/*saT1R2a*/*d*/*e* comparisons). In the GIT (stomach, foregut, midgut and hindgut, Fig. 2B), lower *saT1R* mRNA levels were generally observed when compared to oropharyngeal tissues (note the differing y-axes scales of Fig. 2A versus Fig. 2B). The *saT1R3* gene was significantly higher expressed than the remaining T1R gene-set in both midgut (*P* < 0.05) and hindgut (*P* < 0.01) segments, while no significant differences were found between *saT1R1*/*saT1R2a*/*d*/*e*/*f* in these 2 regions, and among all *saT1R* in stomach and foregut compartments. In the brain (Fig. 2C), some *saT1R* genes showed remarkable high levels of expression, especially in fore- and hindbrain regions. In the forebrain, *saT1R2d* was the highest expressed gene for all statistical comparisons (*P* < 0.001), followed by *saT1R2e* (*P* < 0.01 and *P* < 0.05, for *saT1R1*/*saT1R2a*/*b*/*R3* and *saT1R2f* comparisons, respectively). In the midbrain, significantly higher expression levels were found for *saT1R2b* when compared to *saT1R2d* (*P* < 0.05), *saT1R1*/*R3* (*P* < 0.01) and *saT1R2e*/*f* (*P* < 0.001), and for *saT1R2a* when compared to *saT1R2e*/*f* transcripts (*P* < 0.05). In the hindbrain, *saT1R2d* was expressed at roughly 20-fold higher levels than *saT1R2e*, and 250- or 2,000-fold higher than *saT1R2f* and *saT1R1*/*saT1R2a*/*b*/*R3*, respectively (*P* < 0.001 for all comparisons); all remaining *saT1R* comparisons were not statistically significant.

### 3.3 WISH studies of *saT1R* in seabream larvae

**Fig. 3:**
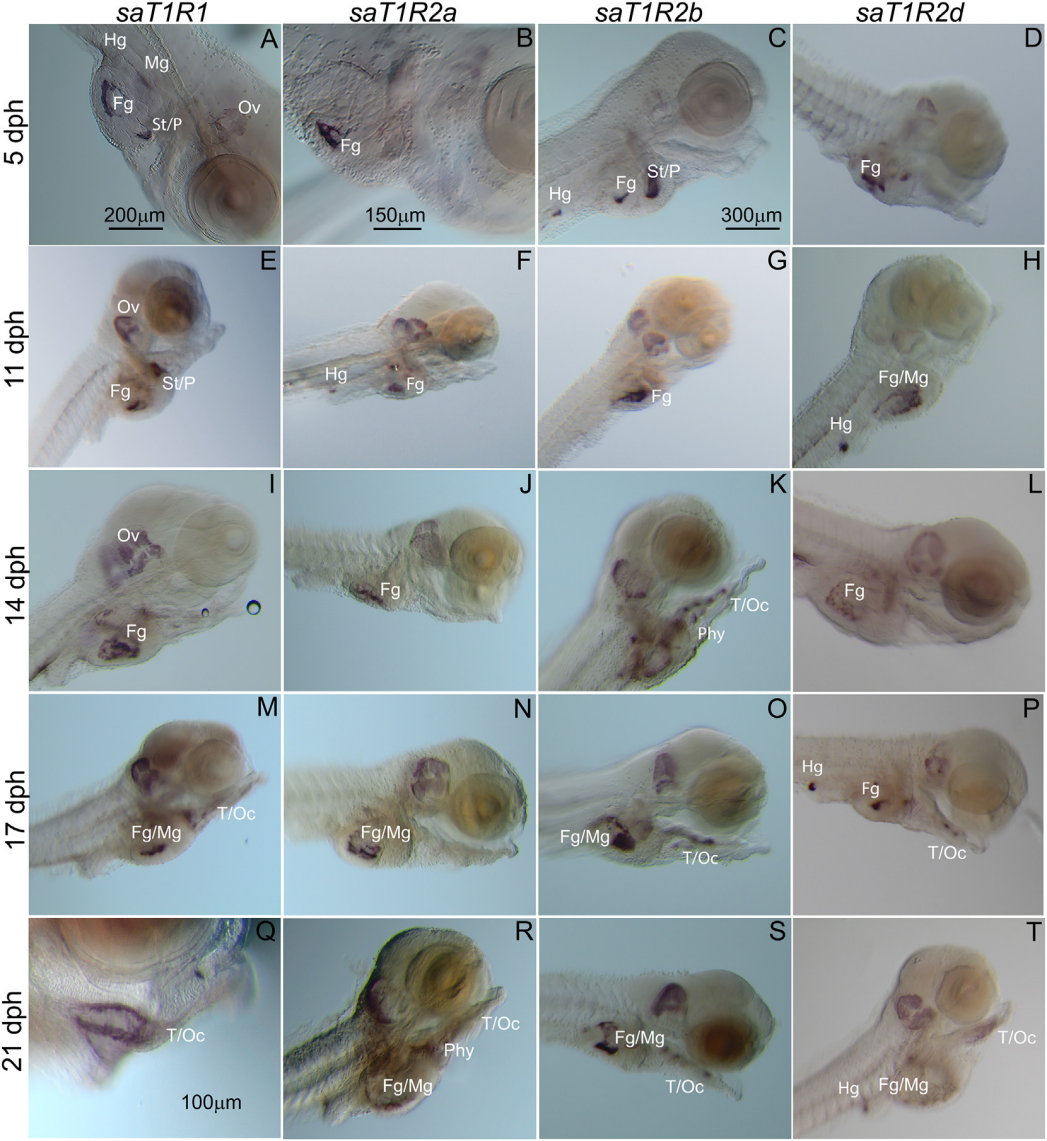
Representative seabream larvae images of the localization of expression for seabream (*sa*) taste 1 receptor (*T1R*) subunits, *saT1R1* (A/O), *saT1R2a* (B/R), *saT1R2b* (C/S) and *saT1R2d* (D/T) genes, at (A–D) 5, (E–H) 11, (I–L) 14, (M−P) 17, and (Q–T) 21 d post hatching determined by the whole-mount mRNA in situ hybridization. Ov = otic vesicle; St = stomach; P = exocrine pancreas; Fg = foregut; Mg = midgut; Hg = hindgut; T = tongue; Oc = oral cavity; Phy = pharynx. Scale bar = 100 μm (Q), 150 μm (B), 200 μm (A, I), 300 μm (C–H, J–P, R**–**T).

**Fig. 4:**
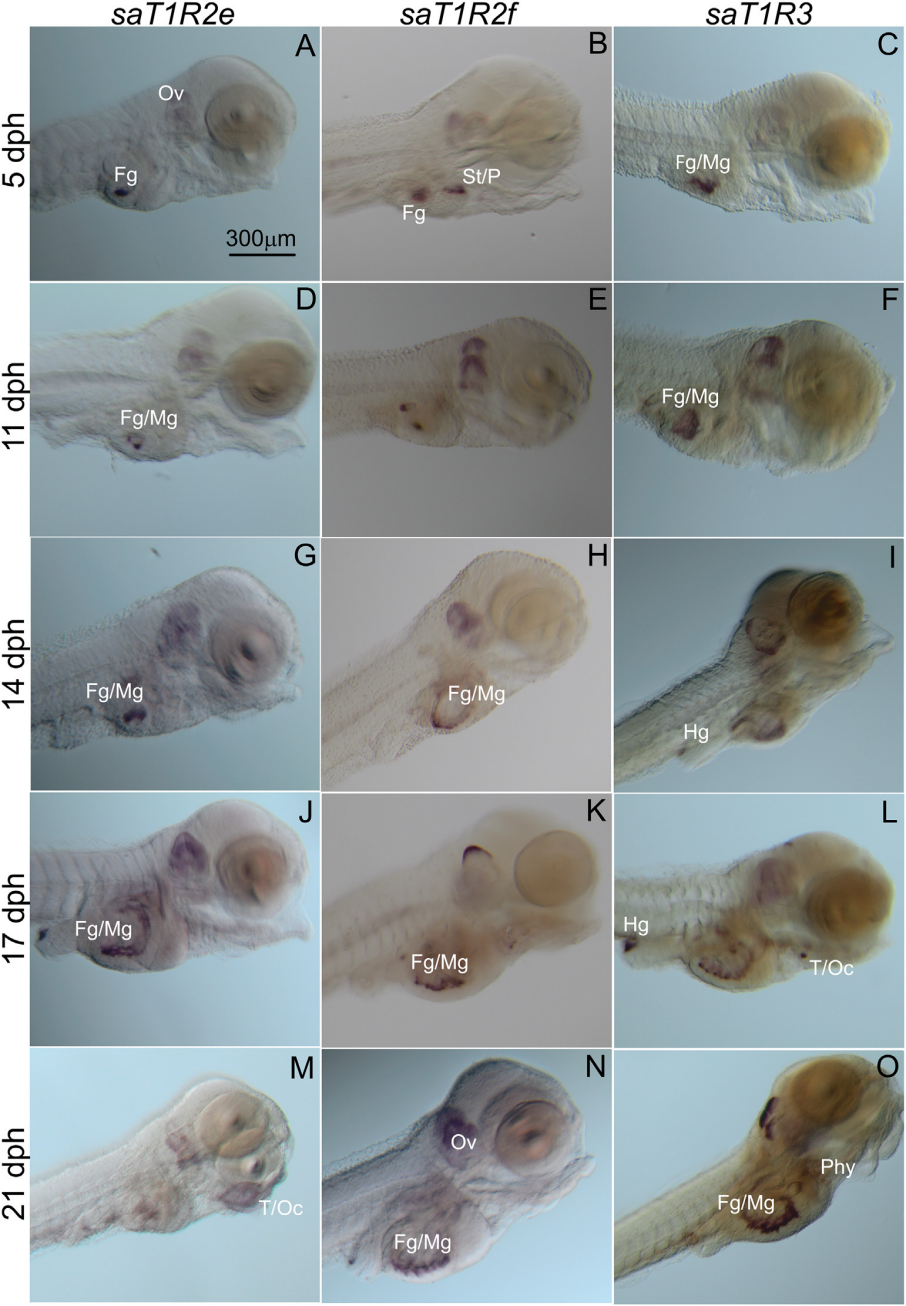
Representative seabream larvae images of the localization of expression for seabream (*sa*) taste 1 receptor (*T1R*) subunits, *saT1R2e* (*A*/*M*), *saT1R2f* (*B*/*N*) and *saT1R3* (*C*/*O*) genes, at (A–C) 5, (D–F) 11, (G–I) 14, (J–L) 17, and (M−O) 21 d post hatching determined by the whole-mount mRNA in situ hybridization analyses. Ov = otic vesicle (Ov); St = stomach (St); P = exocrine pancreas (P); Fg = foregut (Fg); Mg = midgut (Mg); Hg = hindgut (Hg); T = tongue (T); Oc = oral cavity (Oc); Phy = pharynx (Phy). Scale bar = 300 μm (A–O).

**Fig. 5:**
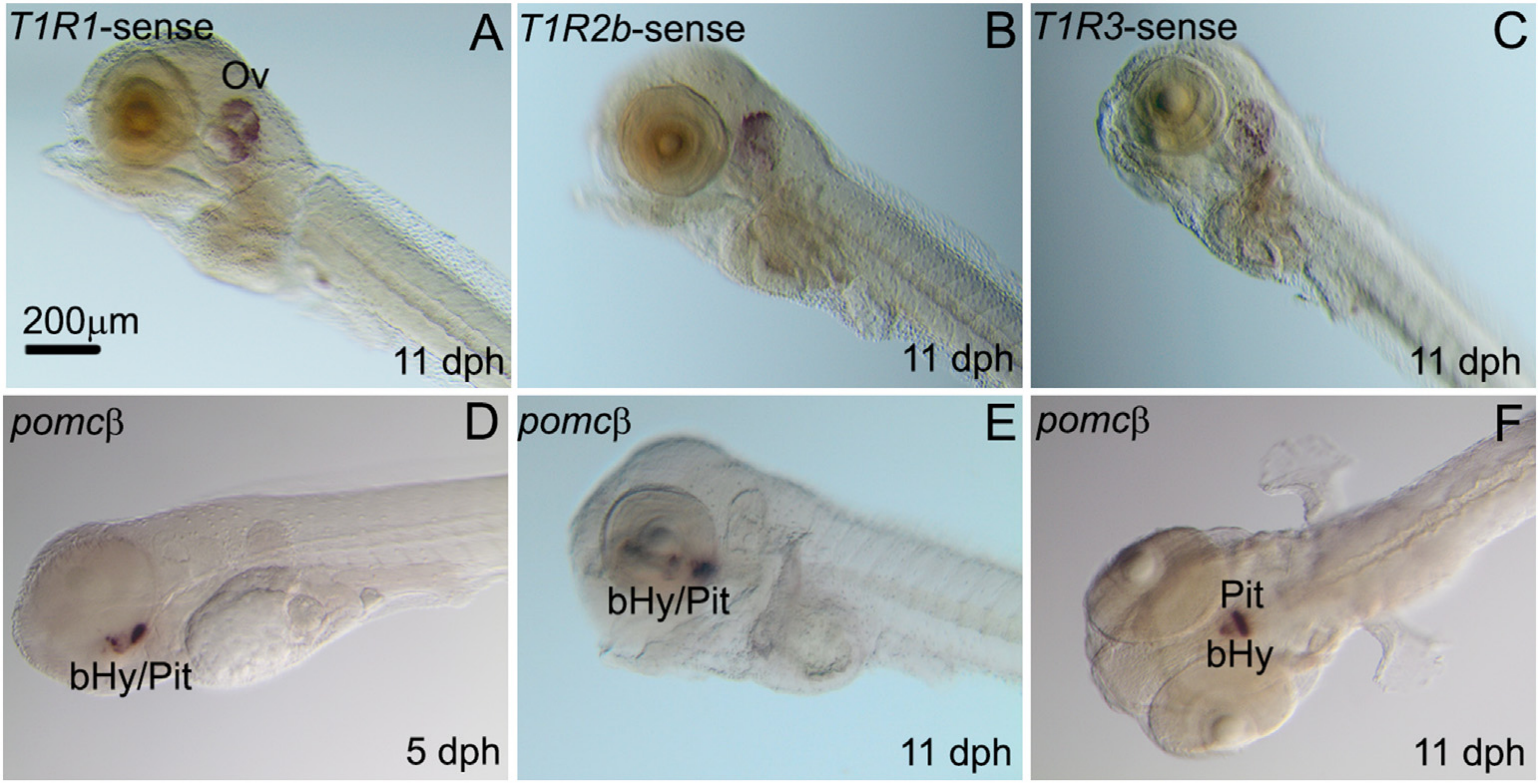
Representative seabream larvae images as determined by the whole-mount mRNA in situ hybridization analyses of negative sense controls for seabream (*sa*) taste 1 receptor (*T1R*) subunits, (A) *saT1R1*, (B) *saT1R2b*, and (C) *saT1R3* at 11 d post hatching (dph), and positive antisense controls using the pituitary/hypothalamic gene marker *proopiomelanocortin β* at (D) 5 and (E, F) 11 dph. Ov = otic vesicle; bHy = basal hypothalamus; Pit = pituitary gland. Scale bar = 200 μm (A-F).

The WISH techniques were used to localize *saT1R* mRNA expression in whole larvae in five selected stages, including yolk-sac nourishment (5 dph) and exogenously feeding (on rotifers) larvae at 4 post-feeding stages (11, 14, 17 and 21 dph). An overview of the *saT1R*'s sites of expression in developing larvae is shown in Fig. 3, Fig. 4, where the anatomical localization is indicated. In pre-feeding larvae, the 7 *saT1R* exhibited overlapping expression patterns in the stomach and the foregut (Fig. 3A–D; Fig. 4A–C), while additional expression in the most posterior region of the intestine was also observed for *saT1R2b* (Fig. 3C). At 11 dph, *saT1R* patterns remained essentially conserved in the 3 developing portions of the gastrointestinal tract (Fig. 3E–H; Fig. 4D–F), and though exogenous feeding had already started, *saT1R* expression was not detected in oral taste tissues (lips, tongue, oral cavity epithelium) for the entire *saT1R* gene set. During post-feeding stages, mRNAs became gradually detected in the oral cavity epithelium and/or tongue and pharynx, while overall maintaining a strong expression in the stomach and intestine. In particular, oral tissue expression was observed for *saT1R2b* at 14, 17 and 21 dph (Fig. 3K, O, S); for *saT1R1*, *saT1R2d* and *saT1R3* at 14 and 17 dph (Fig. 3M, Q; Fig. 3P, T and Fig. 4L, O, respectively) and for *saT1R2e* at 21 dph (Fig. 4M). A summary of gut *vs* oral sites of *saT1R* expression throughout ontogenesis is provided in Table 1. Negative controls using DIG-labeled *saT1R1*/*R2b*/*R3* sense RNA probes were virtually devoid of labeling in gastrointestinal regions, although chromogenic signals were some time detected in the developing otic vesicles (Fig. 5A–C). The cRNA antisense probes synthesized from the pituitary/hypothalamic gene marker *pomcβ* yielded, as expected, well-defined chromogenic signals in the medio-basal hypothalamus (Fig. 5D–F).

**Table 1:** Summary of *saT1R*(*s*) patterns of expression as deduced by whole mount in situ hybridization analyses in gut and oral tissues during larval ontogenesis.

*saT1R(s*)	5 dph	11 dph	14 dph	17 dph	21 dph
*T1R1*	Gut	Gut	Gut	Gut/Oral	Gut/Oral
*T1R3*	Gut	Gut	Gut	Gut/Oral	Gut/Oral
*T1R2a*	Gut	Gut	Gut	Gut	Gut/Oral
*T1R2b*	Gut	Gut	Gut/Oral	Gut/Oral	Gut/Oral
*T1R2d*	Gut	Gut	Gut	Gut/Oral	Gut/Oral
*T1R2e*	Gut	Gut	Gut	Gut	Gut/Oral
*T1R2f*	Gut	Gut	Gut	Gut	Gut

*saT1R* = seabream taste 1 receptor; dph = days post-hatching.

### 3.4 ISH studies of Neurod1, *saT1R*, *saG(i)α1* and *saG(i)α2* in the intestine of adult seabream

This set of experiments was designed to investigate the spatial pattern of gene expressions of *nd1*, *saT1R1*, *saT1R2b*, *saT1R3* and *saG(i)α1-2* in presumptive EECs of the intestine. An additional objective was to investigate the potential co-localization of gene expression in EECs for the following: 1) two of the subunits of heterodimeric complexes forming functional taste receptors (*sa*T1R2/*sa*T1R3), and 2) *saT1R3* (the common subunit of T1R heterodimeric complexes) with either *saG(i)α1* or *saG(i)α2* paralogs, to potentially provide insights into the evolutionary conservation of the *G(i)α*-mediated-taste signal transduction.

**Fig. 6:**
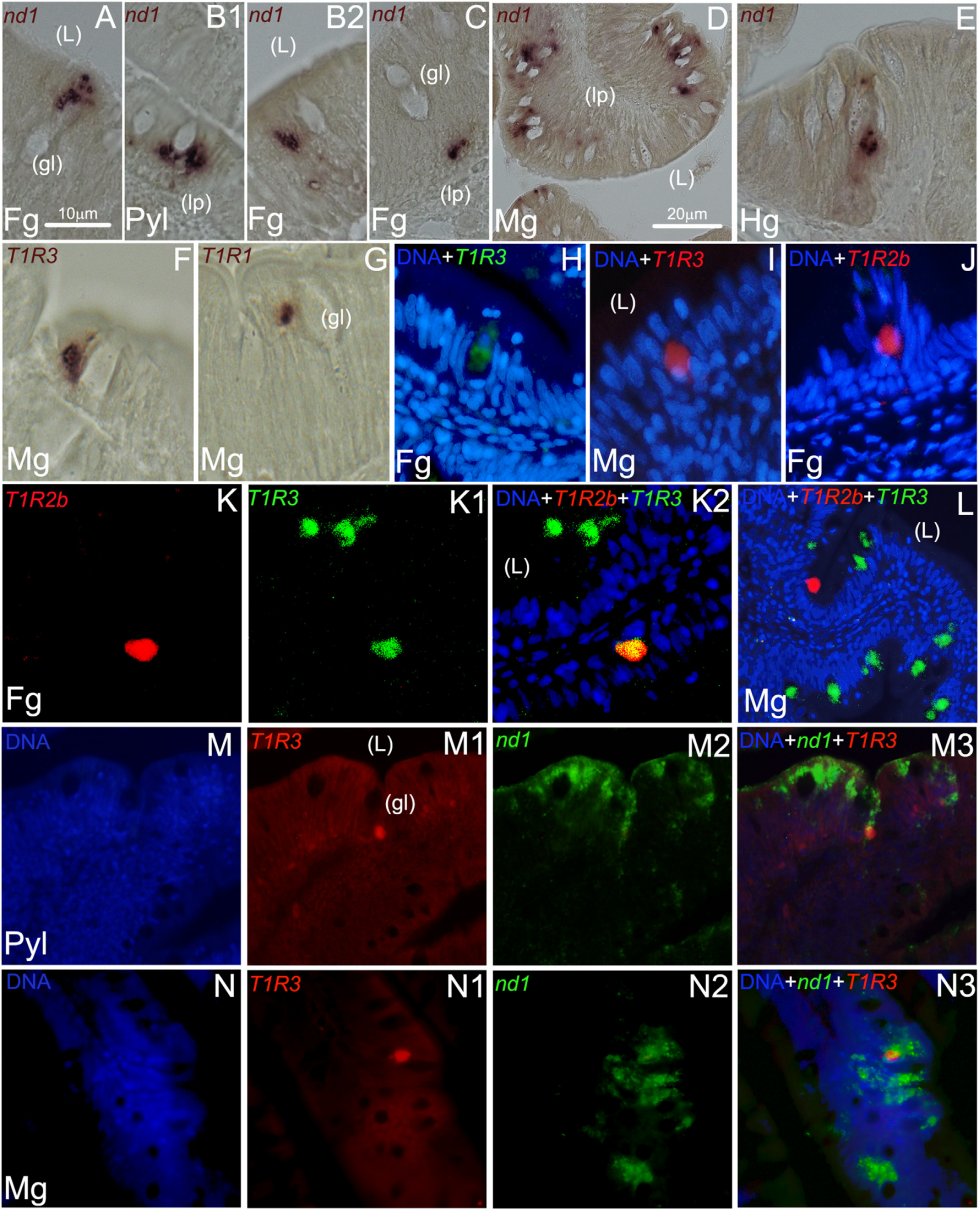
Representative microphotographs of single chromogenic (A–G), single and dual fluorescent (H–J; and K–N3, respectively) in situ hybridization analyses of *nd1*, *saT1R1*, *saT1R2b* and *sa*T1R3 genes in pyloric caeca (Pyl), foregut (Fg), midgut (Mg), and hindgut (Hg) segments of the seabream gastrointestinal tract. Gene names and probe combinations are indicated in the upper left-hand corner of each panel. Signal color corresponds to probe name; Hoechst 33342 (blue) fluorescent dye was used for nuclear DNA counterstain. *nd1* = neurogenic differentiation 1; gl = goblet cells; L = intestinal lumen; lp = lamina propia. Scale bar = 10 μm (A–C, E–K2), 20 μm (D; L–N3).

By using the CISH methods, we first characterized the pattern of expression of *nd1*, a member of the basic-helix-loop-helix (bHLH) family of transcription factors. Transcripts of *nd1* were found throughout the gut portions analyzed, in 3 main locations: 1) in the upper layer of the intestinal mucosa, adjacent to mucus-secreting goblet cells (Fig. 6A), 2) in the intermediate mucosa (Fig. 6B1-B2 and 3) in the bottom layer of the mucosa (Fig. 6C). The *nd1* positive (+) cells were particularly abundant in the midgut, where proliferative outbreaks next to the lamina propia were also identified (Fig. 6D). The *nd1* expression was drastically reduced and essentially restricted to few presumptive EECs in distal (hindgut) segments of the intestine (Fig. 6E).

Based on the previous qPCR analysis, we selected the highest *saT1R* expressed genes in the intestine (*saT1R3*, *saT1R1* and *saT1R2b*) for further detailed investigation. Initial examination by CISH methods showed that they were mostly expressed in the upper mucosal lining, contiguously to goblet cells, exhibiting an expression pattern similar to that shown by a subpopulation of *nd1* (+) cells (Fig. 6F and G). These observations were further corroborated by single-color fluorescent detections using fluorescein-TSA (Fig. 6H; *saT1R3*) and Dig-FastRed (Fig. 6I and J for *saT1R3* and *saT1R2b*, respectively). Next, we investigated whether *saT1R3* and *saT1R2b* gene transcripts were co-expressed in the same presumptive EECs by dual FISH methods, providing evidences that both genes could have either common or independent spatial patterns of expression. Specifically, *saT1R3* (+) cells were by far more abundant than *saT1R2b* (+), and rarely co-expressed *saT1R2b* (Fig. 6L; Fig. 6K-K2). *saT1R2b* was almost always co-expressed with *saT1R3*, although it was occasionally found in presumptive EECs not expressing *saT1R3* (Fig. 6L). To verify the hypothesis that *T1R* genes have, at least partially, nutrient sensing roles in EECs, we next sought to test by dual FISH if, and to what extent, *nd1* (+) EECs also expressed *saT1R3*. Consistently with the CISH experiments reported above, *nd1* (+) cells were mainly found in proximal and medial intestine segments (Fig. 6M2, N2), where they occasionally co-expressed *saT1R3* transcripts (Fig. 6M3, N3).

**Fig. 7:**
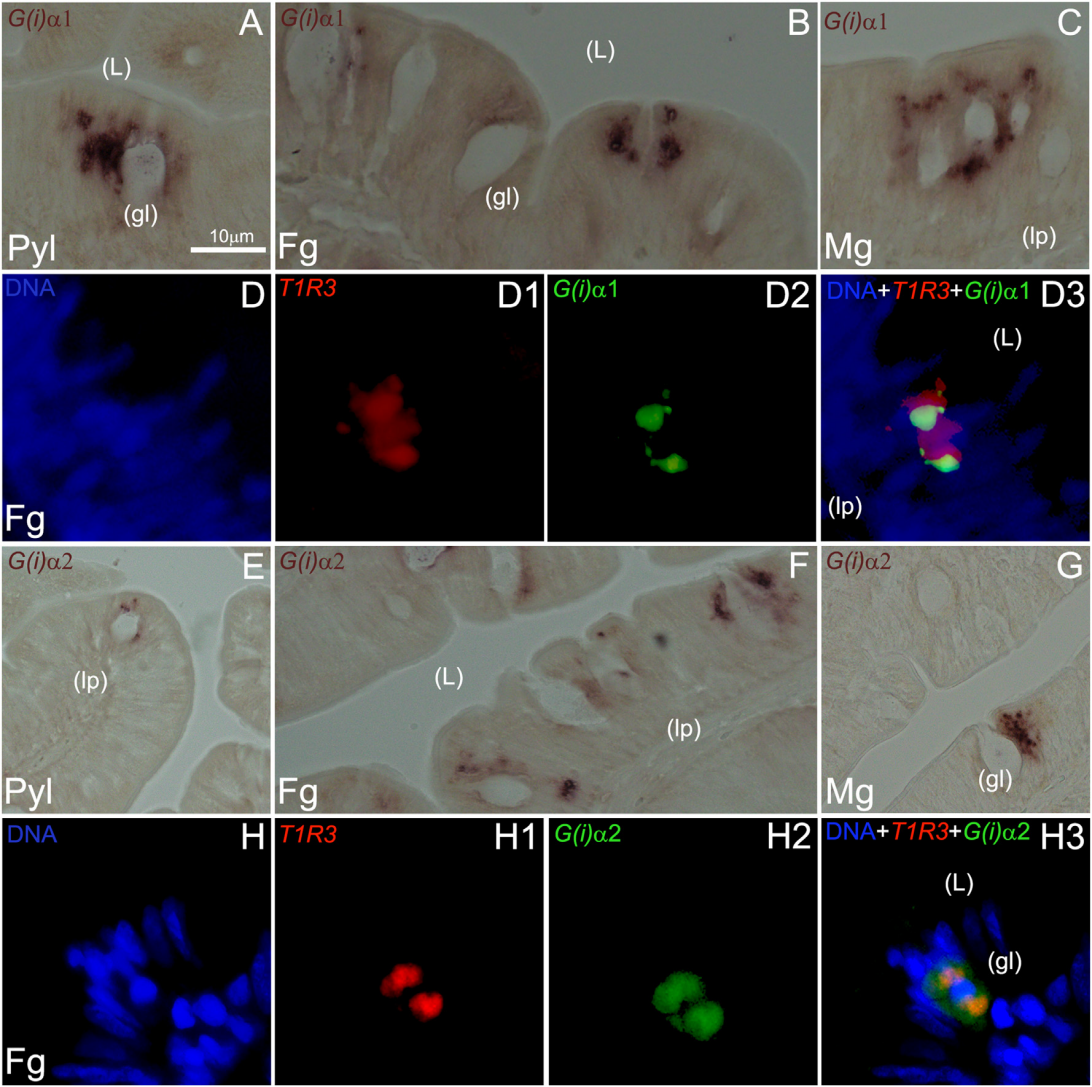
Representative microphotographs of chromogenic and dual fluorescent in situ hybridization analyses of *saG(i)α1* (A–C and D–D3, respectively) and *saG(i)α2* (E–G and H–H3, respectively) genes in pyloric caeca (Pyl), foregut (Fg) and midgut (Mg) segments of the seabream gastrointestinal tract. Gene names and probe combinations are indicated in the upper left-hand corner of each panel. Signal color corresponds to probe name. gl = goblet cells (gl); L = intestinal lumen (L); lp = lamina propia (lp) Scale bar = 10 μm (A–H3).

Furthermore, we conducted a series of ISH experiments to characterize the spatial expression patterns of the 2 gene paralogs *saG(i)α1* and *saG(i)α2* by single CISH methods. Both transcript-types were found abundantly expressed in a widespread fashion resembling the 3 main spatial domains previously described in this study for the EEC-marker *nd1* in pyloric, fore- and midgut segments (Fig. 7A–C and Fig. 7E–G for *saG(i)α1* and *saG(i)α2*, respectively). In the hindgut, *saG(i)α1* and *saG(i)α2* expressing cells were less frequent (data not shown). When dual FISH was employed for both *saG(i)α1*/*saT1R3* and *saG(i)α2*/*saT1R3* probe combinations, several cases of co-localization in *saT1R3* (+) EECs with both *saG(i)α* *1-2* transcripts were observed (Fig. 7D-D3 and Fig. 7H-H3 for *saG(i)α1*/*saT1R3* and *saG(i)α2*/*saT1R3* combinations, respectively).

### 3.5 ISH studies of *ghr*, *cck*, *pyy* and *pg* hormone genes and their colocalization with the *saT1R3* subunit in the intestine of adult seabream

**Fig. 8:**
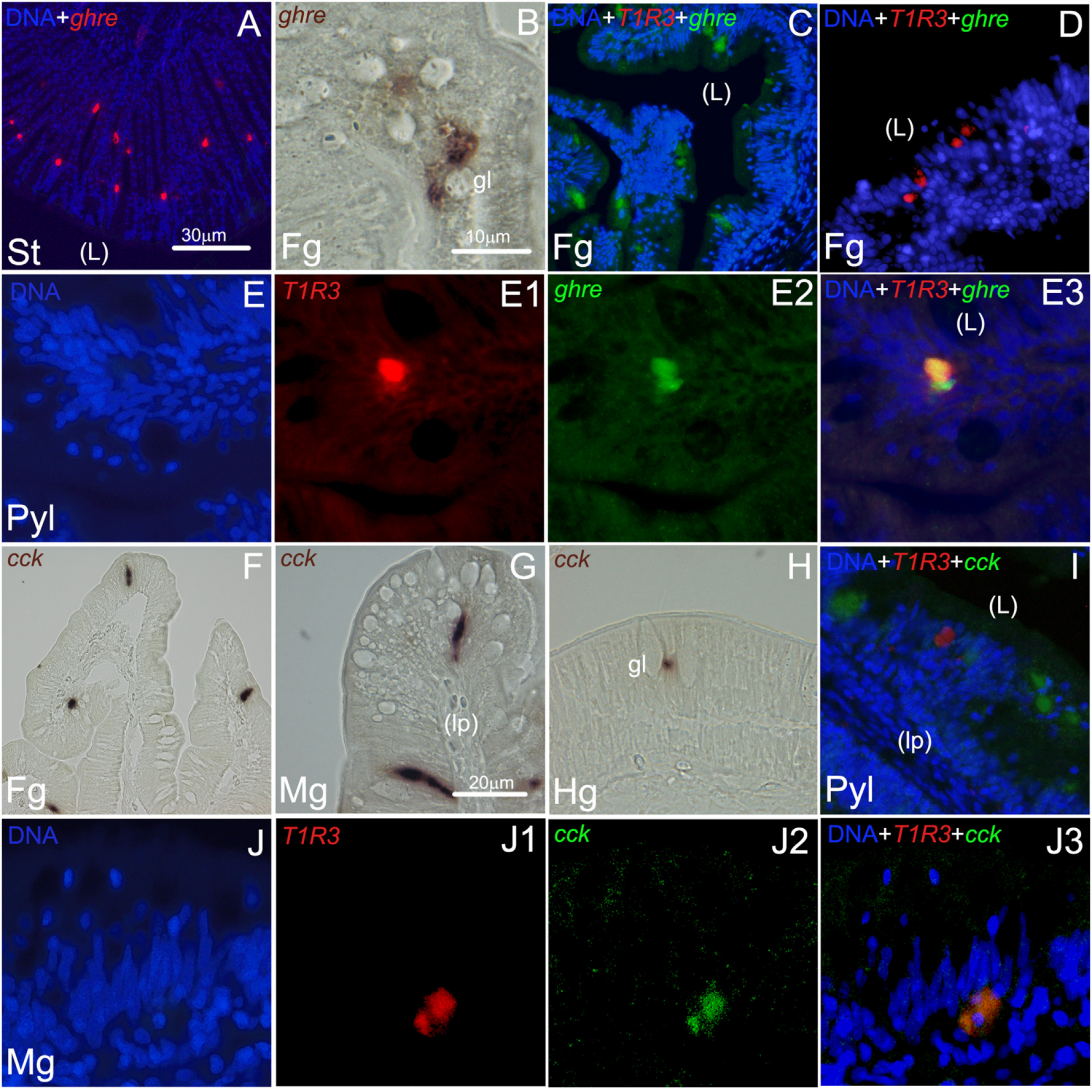
Representative microphotographs of chromogenic (B, F–H), single and dual fluorescent in situ hybridization (A and C–E3/I–J3, respectively) of *ghre* and *cck* genes in stomach (St), pyloric caeca (Pyl), foregut (Fg), midgut (Mg), and hindgut (Hg) segments of the seabream gastrointestinal tract. Gene names and probe combinations are indicated in the upper left-hand corner of each panel. Signal color corresponds to probe name. *ghre* = ghrelin; *cck* = cholecystokinin; gl = goblet cells (gl); L = intestinal lumen; lp = lamina propia (lp). Scale bar = 10 μm (B, E–E3; J–J3), 20 μm (G–I), 30 μm (A, C–D; F).

In another set of ISH experiments, we aimed to provide direct evidence for mRNAs co-expression of the gut peptides *ghr*, *cck*, *pyy* and *pg* with *saT1R3* gene, to support the hypothesis that gut hormone secretion upon T1R-mediated gut sensing might also occur in fish. Using cRNA probes we found that *ghr* was abundantly expressed within scattered cells in the gastric mucous membrane (Fig. 8A); this first experiment was intended to test the effectiveness of our protocol in a tissue where this gene is known to be highly expressed. When using intestinal tissues, *ghr* (+) cells were also clearly identified in presumptive EECs of the proximal intestine by CISH (Fig. 8B). Dual FISH in this area showed several *ghr* (+) cells that did not co-express *saT1R3* (Fig. 8C), as well as *saT1R3* (+) cells devoid of *ghr* expression (Fig. 8D). However, a few cases of *ghr*/*saT1R3* co-localization were also identified (Fig. 8E-E3). No evidence of *ghr* expression was detected in distal portions of the gut (not shown).

**Fig. 9:**
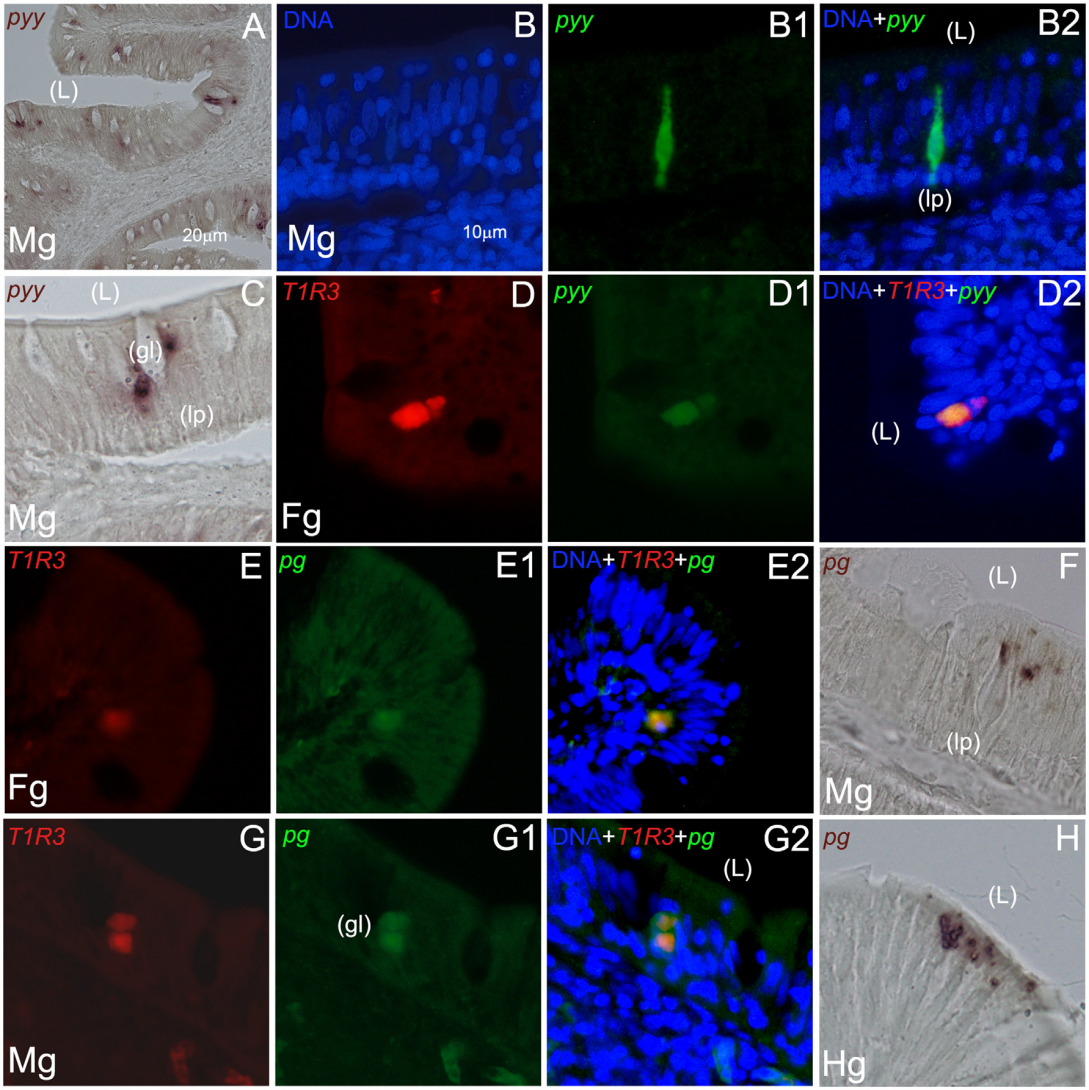
Representative microphotographs of chromogenic (A, C, F, and H), single (B–B2) and dual fluorescent in situ hybridization (D–D2; E–E2; G–G2) of *pyy* and *pg* genes in foregut (Fg), midgut (Mg), and hindgut (Hg) segments of the seabream gastrointestinal tract. Gene names and probe combinations are indicated in the upper left-hand corner of each panel. Signal color corresponds to probe name. *pyy* = peptide YY; *pg* = proglucagon; gl = goblet cells; L = intestinal lumen; lp = lamina propia. Scale bar = 10 μm (B–H), 20 μm (A).

Next, we analyzed *cck* mRNA localization throughout the intestine, initially by CISH, and found a particularly high density/abundance of transcripts, as deduced by the intense chromogenic staining. A strikingly elongated *cck* cell morphology was observed in proximal (Fig. 8F and G), but not in distal (Fig. 8H) gut segments. Dual FISH experiments using *cck* and *saT1R3* cRNA probes revealed that the 2 targets largely exhibited independent spatial domains (Fig. 8I), although some cases of co-localization were also observed (Fig. 8J–J3). Likewise, *pyy* and *pg*-expressing EECs were also found along intestinal segments, although usually with low abundance. Particularly, flask shaped *pyy* (+) cells were clearly identified in the midgut, by both CISH (Fig. 9A, C) and green-FISH (Fig. 9B-B2), and additionally in the foregut, where a few cases of co-localization with *saT1R3* transcripts were observed (Fig. 9D–D2). The *pg* transcripts were even more rarely detected than those of *pyy*. Indeed, *pg* (+) cells were only sparsely noticed, with no clear concentration or pattern (Fig. 9F), although few cases of co-localization of expression with *saT1R3* were also observed (Fig. 9E-E2 and Fig. 9G-G2, in fore- and midgut respectively). Additionally, *pg* transcripts were identified in presumptive EECs of the hindgut, although clear ISH signals were only visible by CISH methods (Fig. 9H).

## 4 Discussion

In a recent study, we reported the complete *T1R* gene repertoire of gilthead seabream, consisting of eight members including *saT1R1*, *saT1R3* and six *saT1R2* (*a-f*), and functionally characterized the in vitro responses of a subset of heterodimers, namely *saT1R1*/*R3*, *saT1R2a*/*R3* and *saT1R2b*/*R3*, to L-amino acids and sweet ligands (Angotzi et al., 2020). Here, we further explored the mRNA expression profiles of all *saT1R* in both larval and adult tissues. During early larval development, mRNA levels were quantified in whole-body of larval stages encompassing the transition from endogenous to first exogenous feeding (initiated at 9 dph). The entire *T1R* gene repertoire was expressed from 1 dph onwards, without significant variations until 10 dph. At 12 dph, as soon as first exogenous food is digested and metabolized, all *saT1R* transcripts, and especially *saT1R2b*, significantly increased their expression levels. The highest level of expression and abrupt rise of *saT1R2b* expression following first exogenous feeding and its earlier appearance in oropharyngeal tissues as deduced by the whole mount in situ analyses, suggests that this paralog may be playing major roles related to feeding. In support of this hypothesis, *saT1R2b/R3* was the most responsive and sensitive heterodimer to L-amino acid stimulations in this species (Angotzi et al., 2020). Paradoxically, the *saT1R3* gene encoding the shared subunit of receptors signaling both sugar (T1R2/T1R3) and protein (T1R1/T1R3) rich foods in mammals (Roper, 1989; Hoon et al., 1999; Adler et al., 2000; Yarmolinsky et al., 2009), and responding to a wide spectrum of L-amino acids (T1R1/T1R3 and T1R2_n_/T1R3) in fish (Oike et al., 2007), was among the lowest expressed gene throughout the larval stages analyzed. It is believed that alternative dimeric arrangements among T1R subunits might also potentially occur in cell membranes, including homodimerization among the highest expressed *sa*T1R subunits (Damak et al., 2003; Herness, 2018), albeit these types of protein combinations have mainly been reported for the T1R3 homodimer (Masubuchi et al., 2013; Kojima et al., 2014; Lee and Cohen 2015; Mafi et al., 2021). Therefore, the reason behind these unexpected differences in gene expression levels remains elusive at present.

The gene expression profiling during early development was extended (up to 21 dph) through mRNA localization studies by whole-mount ISH. Surprisingly, the results revealed that at the earliest larval stages analyzed (5 and 11 dph), the entire set of *saT1R* transcripts were virtually confined to the developing GIT. Expression of *saT1R* in oropharyngeal regions was detected at 14 dph for *saT1R2b*, and gradually followed by the other *saT1R* members, except *T1R2f* whose expression was only observed in the GIT at 21 dph. Altogether, these findings suggest that *saT1R* expression during early larval stages spanning first exogenous feeding arise heterochronously, and that extraoral (gastrointestinal) taste-related perception occurs earlier than the buccal/intraoral perception. However, starting at 14 dph onwards both tissue modalities coexist. The observed “delayed” onset of *saT1R* expression in oropharyngeal tissues is consistent with the notion that, in most fish, functional taste buds and the capability to efficiently perceive gustatory qualities might arise later in development, although morphologically mature taste buds appear just before or at the onset of exogenous feeding in some species (Hansen et al., 2002; Kasumyan, 2001). Marine fish larvae, in particular, are considered primarily visual feeders, with olfaction and the lateral line providing additional inputs to visually oriented feeding (Rønnestad et al., 2013). For instance, the study of the development of sensory systems in sharpsnout seabream (*Diplodus puntazzo*) larvae showed that at the start of exogenous feeding larvae rely on olfactory ciliated and microvillate sensory cells and free cephalic neuromasts, besides vision, to detect the presence of food, while intraoral and extraoral taste buds only differentiated in the post-larval stage at 48 and 51 dph, respectively (Boglione et al., 2003). Accordingly, behavioral experiments testing agar pellets indicated that fish larvae at first feeding respond only to a limited number of taste stimuli, but as larval development progresses, the spectrum and effectiveness of amino acid perception greatly increases (Hughes, 1991, 1993; Kasumyan and Sidorov, 2005; Kasumyan, 2001).

The biological significance of *saT1R* expression in lecithotrophic stages undergoing organogenesis of the gastrointestinal tract is unclear considering that larval development and nutrition at this point are fueled by the endogenous energy supplies of the yolk. The transition period from exhaustion of yolk reserves to the onset of exogenous feeding is a critical step for larval survival, and has been associated with massive mortalities depending on the fish species (Yúfera et al., 2005; Palazzi et al., 2006). Successful transition to exotrophic life stages largely relies on the functional maturation of the gastrointestinal structures necessary for food digestion and absorption, alongside the development of efficient signaling pathways through which the brain and the gastrointestinal system communicate to regulate food intake and energy homeostasis. Newly hatched larvae have a rudimentary tubular intestine, but the intestinal mucosa starts to increase its thickening and folding concomitantly with mouth opening (i.e., few days before the transition to exogenous feeding). At this point, enterocytes initiate their apical differentiation by developing a brush border microvillus membrane that becomes fully functional at approximately the third week post-hatching (Cahu and Zambonino-Infante, 1994; Moyano et al., 1996; Ribeiro et al., 1999; Zambonino-Infante et al., 2008). Despite the low degree of morphological differentiation, at mouth opening most fish species investigated so far, including marine (Zambonino-Infante and Cahu, 2001; 2008; Rønnestad et al., 2013; Yúfera and Darias, 2007) and freshwater (Lahnsteiner, 2017) species, have active cytosolic (intracellular; e.g., leucine–alanine peptidases) and secretory (pancreatic, trypsins, lipases and amylases) proteolytic, lipolytic, and carbohydrate splitting enzymes in their intestines. These findings suggest that the onset of digestive enzyme expression and activity is not induced by food but rather genetically hardwired. Similarly, mRNA transcripts coding for endocrine hormones and neuropeptides of the brain-gut axis have been reported prior to the onset of exogenous feeding, including *ghr*, *cck*, neuropeptide Y, *pomc*, cocaine-amphetamine-regulated transcript and prepro-orexin (Hoang et al., 2016; Ping et al., 2014; Kurokawa et al., 2000). In line with these observations, it is reasonable to postulate that the early (pre-feeding) onset of the molecular machinery underlying *saT1R* expression in the gastrointestinal tract might be an anticipatory and genetically programmed mechanism, gradually yielding functional saT1R proteins to possibly exert chemosensory roles in the gut of older larvae and adult fish.

Next, we sought to ascertain the distribution of *saT1R* mRNA transcripts in different adult fish tissues, to establish possible similitudes with mammalian vertebrates, in which T1R are widely expressed in body tissues, where they perform chemosensory functions beyond oral taste sensation (Finger and Kinnamon, 2011). Hence, gene expression was quantified by qPCR in different tissues within oropharingeal, GIT and brain regions. Our findings are in accordance with previous studies that reported several taste receptors and taste signaling components in the GIT of different fish species (Polakof and Soengas, 2013; Latorre et al., 2013; Ronnestad et al., 2016; Yuan et al., 2020; Calo et al., 2021; Kinnamon, 2012; Morais, 2017), reinforcing the hypothesis that the T1R-mediated gut sensing mechanisms could be conserved throughout vertebrate evolution. The significantly higher levels of *saT1R3* expression observed in oropharyngeal and GIT tissues suggest that this gene might be locally demanded at higher transcriptional rates due to heterodimerization with *saT1R1* or *saT1R2* subunits, since *saT1R3*/*saT1R3* homodimers do not seem to respond to L-amino acids stimulations in this species (Angotzi et al., 2020). However, the existence of T1R3 homo-oligomers cannot be dismissed since these have been described in mammals and proposed to sense calcium and magnesium taste (Nelson et al., 2001). Interestingly, there were significantly higher mRNA levels of both *saT1R2b* and *saT1R2f* than *saT1R2a*/*T1R2d*/*T1R2e* in gills. These gene expression patterns are in agreement with those recently described for *T1R2b*/*e* ortholog counterparts in grass carp (*Ctenopharyn godonidellus*) (Yuan et al., 2020), suggesting that the T1R2-mediated chemosensing functions may have been highly retained in gills throughout teleost radiation. We found also high levels of expression for the paralog subtypes *saT1R2d*/*e* and *saT1R2d*/*e*/*f* in forebrain and hindbrain, respectively, suggesting that these genes might have a tissue-specific chemosensory role in these brain compartments. In accordance, specialized glucose-sensing neurons mainly located in paraventricular and arcuate nuclei of the hypothalamus and in the nucleus of the solitary tract of the brainstem, are known to regulate extracellular glucose concentration through sweet taste-like signaling in murine models (Ren et al., 2009; Herrera Moro Chao, 2016; McCaughey, 2021). Similarly, the T1R nutrient-sensing functions with implications on food intake have also been described in some brain regions of rainbow trout (*Oncorhynchus mykiss*) (Otero-Rodiño et al., 2015; Comesaña et al., 2018a; 2018b).

In the present study, we additionally designed a comprehensive set of experiments with the purpose of describing the spatial pattern of expression of *saT1R1*, *saT1R2b*, *saT1R3*, *saG(i)α1* and *saG(i)α2* genes, and to substantiate possible mRNA co-expression of the *saT1R3* subunit with both *saG(i)α1-2* and with selected gut hormones (*ghr*, *cck*, *pyy* and *pg*) in presumptive EECs along the intestine. In both mammalian and fish models, it has been previously shown that the bHLH transcription factor *nd1* plays essential roles to direct intestinal progenitor cells to an EEC fate, and that it is selectively expressed in this GI cell population (Li et al., 2011, 2019; Ye et al., 2019). Therefore, we first identified presumptive EECs employing *nd1* as a specific EEC-marker, and found that it is expressed in cells located in different layers of the intestinal mucosa, possibly reflecting a continuous epithelial renewal in spatially distinct compartments (Sun et al., 2018). EECs represent a small population of scattered and highly specialized gut epithelial cells that respond to luminal contents, acting as chemoreception units capable of releasing signaling factors (Raybould, 2010; Young, 2011). Their sensory properties are exerted by different nutrient and non-nutrient-sensing receptors, mainly GPCRs, implicated in the perception of glucose, amino acids, fatty acids, bile acids, phytochemicals or secondary products derived from microbial fermentation (Gribble and Reimann, 2019). We demonstrated that *saT1R* genes are mostly expressed in presumptive mature EECs located in the upper epithelial lining of the intestinal mucosa, and that *saT1R3* (+) cells are usually found lying contiguously to mucus secreting goblet cells, often in contact with the gastrointestinal lumen. In addition, *sa*T1R3 (+) EECs were also detected near the base of the lamina propria membrane, apparently without reaching the intestinal lumen. Although the interpretation of this cell patterning heterogeneity is challenging, it correlates well with the “open” or “closed” EEC-types described in higher vertebrates to sense gut contents either directly (open-type), or indirectly (closed-type) through neural or humoral pathways (Sternini, 2007; Latorre et al., 2016).

The examination of overlapping expressions of *saT1R2b*/*R3* by dual FISH revealed the existence of a greater population of *saT1R3* (+) cells whose major fraction did not express *saT1R2b*, while the latter was almost always co-expressed with *saT1R3*. Similar mutual distributions of the single T1R subunit components have been previously described in both oral (Nelson et al., 2002; Li, 2009) and gastrointestinal (Daly et al., 2013) tissues of mammals, and in oral tissues of fish (Oike et al., 2007). However, this study provides for the first time in situ morphological evidence that heterodimerization is likely an evolutionary preserved mode of taste receptors coupling in fish gut sensing. While the presence of *saT1R3* (+) cells that did not express *saT1R2b* (+) is consistent with the existence of different *T1R3* (+) subpopulations selectively co-expressing one (or more) *T1R* subunits (Oike et al., 2007), the functional significance of the small fraction of *saT1R2b* (+) cells devoid of *saT1R3* expression is yet to be explained, and again suggests that this receptor subunit could additionally function as a monomer or homodimer (Herness, 2018).

Since the first report uncovering the lack of Gα-gust orthologs in fish genomes (Oka and Korsching 2011; Ohmoto et al., 2011), other Gαi subunits have been proposed to mediate taste signal transduction, and studies using immunoreactive and quantitative molecular assays further documented Gαi expression in the GIT of some fish species (Latorre et al., 2013; Calo et al., 2021). Here, we provided clear evidence that *saG(i)α1* and *saG(i)α2* genes are highly expressed in the proximal GIT, with spatial expression patterns resembling those previously described for the EEC marker *nd1*. Through dual FISH assays, we further demonstrated that both *saG(i)α* genes are expressed in EECs potentially implicated in *sa*T1R3-mediated molecular sensing, thus supporting their functional homology to Gα-gust as intracellular taste-like transducer(s) in the GIT (Bertrand, 2009; Young, 2011; Angotzi et al., 2020).

Mammalian EECs are known to produce several peptides, and have been traditionally classified according to the hormones they secrete. The best characterized EECs are the X/A-cells (in mice) or P/D1-cells (in humans) producing GHR, L-type cells producing glucagon-like peptides 1 and 2 and peptide YY (PYY), the I-type cells producing cholecystokinin (CCK), and the K-type cells producing glucose-dependent insulinotropic polypeptide (Sjölund et al., 1983). Furthermore, different EEC subsets can overlap in the co-expression of multiple hormones (Habib et al., 2012; Latorre et al., 2016; Fothergill and Furness, 2018). To verify assumptions based on mammalian studies that T1R might function as EEC-sensory transducers (Burman and Kaji, 2021) in fish, we aimed to determine whether the *saT1R3* gene was expressed in fish-like X/A- L-, or I- specialized EEC types. Using dual FISH methods, we identified some cases of co-localization between *saT1R3* and *ghr*, *cck*, *pyy* and *pg* genes in different regions of the GIT, as well as independent and non-overlapping expression domains. While these spatially correlated patterns of expressions corroborate a plausible direct role for *sa*T1R as nutrient-sensing targets regulating hormone secretion in seabream, the identification of additional *saT1R3* (+) cell-subsets devoid of endocrine peptides expression could indicate that *sa*T1R-mediated chemosensing functions might occur in these tissues via mechanisms that are both dependent and independent of endocrine pathways. Indeed, in addition to their potential function as nutrient sensors participating in food digestion, nutrient absorption and metabolism, mammalian T1R, together with T2Rs, have been proposed to also regulate gut innate immune responses to compounds secreted by microbial pathogens (Lee and Cohen 2015; Triantafillou et al., 2018). On the other hand, putative L-, K- or I- EEC types that did not express *sa*T1R3 might potentially be equipped with other nutrient sensors such as extracellular calcium sensing receptors, taste variants of metabotropic glutamate receptors and free fatty acid receptor 2/3, among others (Raka et al., 2019; Burman and Kaji, 2021).

Interestingly, single-cell RNAseq surveys of the murine small intestine recently identified a broader set of genes for different epithelial cell lineages (goblet, Paneth, or tuft cells), including Krüppel-like factors (KLf3-6), mucosal pentraxin 2, and epithelial cytokines (thymic stromal lymphopoietin and leukocyte common antigen) (Haber et al., 2017). In the current work, the employment of seabream orthologs to these gene markers would have contributed to the characterization of diverse epithelial cell types potentially present in the gut of fish. Unfortunately, many of these markers have not yet been annotated in the seabream genome, and therefore it was not feasible to perform such type of cellular screening. Finally, drawbacks related to practical aspects of fluorescent imaging procedures (variability among experiments and photobleaching, among others), coupled to the remarkably low number of identified *saT1R3*+ cells, hampered our efforts to acquire reliable quantitative estimations of gut hormone + cells co-expressing *saT1R3*. Future work in fish model species that are amenable to a dual reporter transgenic approach in vivo, would further verify the hypothesis of T1R chemosensory roles in the regulation of fish digestive processes put forward in this study.

## 5 Conclusions

Altogether, these findings provide new information on the T1R-mediated chemosensing capabilities in the GIT of a carnivorous fish species, and suggest a likely evolutionarily conserved role for *sa*T1R as nutrient-sensors modulating gut hormone secretion. Furthermore, our data support the hypothesis that the *sa*T1R-mediated gut sensing mechanisms might occur at least partially, through the involvement of the sensory transducers *saG(i)α1* and *saG(i)α2*, thus validating their functional homology to the mammalian *G(i)α* subunit gustducin as taste-like intracellular components in the GIT of fish.

## Author contributions

**Anna Rita Angotzi**: Methodology, Investigation, Formal analysis, Writing - Original Draft, Visualization, Writing - Review & Editing. **Esther Leal**: Investigation, Writing - Review & Editing. **Sara Puchol**: Investigation, Writing - Review & Editing. **Jose Miguel Cerdá-Reverter**: Conceptualization, Methodology, Formal analysis, Writing - Review & Editing, Supervision, Project administration, Funding acquisition. **Sofia Morais**: Conceptualization, Writing - Review & Editing, Funding acquisition.

## Declaration of Competing Interest

We declare that we have no financial and personal relationships with other people or organizations that can inappropriately influence our work, and there is no professional or other personal interest of any nature or kind in any product, service and/or company that could be construed as influencing the content of this paper.

## References

[bib1] Adler E., Hoon M.A., Mueller K.L., Chandrashekar J., Ryba N.J., Zuker C.S. (2000). A novel family of mammalian taste receptors. Cell.

[bib2] Ahmad R., Dalziel J.E. (2020). G Protein-coupled receptors in taste physiology and pharmacology. Front Pharmacol.

[bib3] Alpers D.H. (2010). Nutrient sensing in the gastrointestinal tract. Curr Opin Gastroenterol.

[bib4] Angotzi A.R., Puchol S., Cerdá-Reverter J.M., Morais S. (2020). Insights into the function and evolution of taste 1 receptor gene family in the carnivore fish gilthead seabream (*Sparus aurata*). Int J Mol Sci.

[bib5] Baldwin M.W., Ko M.C. (2020). Functional evolution of vertebrate sensory receptors. Horm Behav.

[bib6] Bertrand P.P. (2009). The cornucopia of intestinal chemosensory transduction. Front Neurosci.

[bib7] Boglione C., Giganti M., Selmo C., Cataudella S. (2003). Morphoecology in larval fin-fish: a new candidate species for aquaculture, *Diplodus puntazzo* (Sparidae). Aquacult Int.

[bib8] Braun T., Mack B., Kramer M.F. (2011). Solitary chemosensory cells in the respiratory and vomeronasal epithelium of the human nose: a pilot study. Rhinology.

[bib9] Burman A., Kaji I. (2021). Luminal chemosensory cells in the small intestine. Nutrients.

[bib10] Cahu C.L., Zambonino-Infante J.L. (1994). Early weaning of sea bass (Dicentrarchus labrax) larvae with a compound diet: effect on digestive enzymes. Comp Biochem Physiol.

[bib11] Calo J., Blanco A.M., Comesaña S., Conde-Sieira M., Morais S., Soengas J.L. (2021). First evidence for the presence of amino acid sensing mechanisms in the fish gastrointestinal tract. Sci Rep.

[bib12] Chandrashekar J., Hoon M.A., Ryba N.J.P., Zuker C.S. (2006). The receptors and cells for mammalian taste. Nature.

[bib13] Comesaña S., Velasco C., Ceinos R.M., López-Patiño M.A., Míguez J.M., Morais S., Soengas J.L. (2018). Evidence for the presence in rainbow trout brain of amino acid-sensing systems involved in the control of food intake. Am J Physiol Regul Integr Comp Physiol.

[bib14] Comesaña S., Velasco C., Conde-Sieira M., Míguez J.M., Soengas J.L., Morais S. (2018). Feeding stimulation ability and central effects of intraperitoneal treatment of L-Leucine, L-Valine, and L-Proline on amino acid sensing systems in rainbow trout: implication in food intake control. Front Physiol.

[bib16] Daly K., Al-Rammahi M., Moran A., Marcello M., Ninomiya Y., Shirazi-Beechey S.P. (2013). Sensing of amino acids by the gut-expressed taste receptor T1R1-T1R3 stimulates CCK secretion. Am J Physiol Gastrointest Liver Physiol.

[bib17] Damak S., Rong M., Yasumatsu K., Kokrashvili Z., Varadarajan V., Zou S., Jiang P., Ninomiya Y., Margolskee R.F. (2003). Detection of sweet and umami taste in the absence of taste receptor T1r3. Science.

[bib18] Depoortere I. (2014). Taste receptors of the gut: emerging roles in health and disease. Gut.

[bib19] Dyer J., Salmon K.S.H., Zibrik L., Shirazi-Beechey S.P. (2005). Expression of sweet taste receptors of the T1R family in the intestinal tract and enteroendocrine cells. Biochem Soc Trans.

[bib20] Finger T.E. (2005). Cell types and lineages in taste buds. Chem Senses.

[bib21] Finger T.E., Kinnamon S.C. (2011). Taste isn't just for taste buds anymore. F1000 Biol Rep.

[bib22] Fothergill L.J., Furness J.B. (2018). Diversity of enteroendocrine cells investigated at cellular and subcellular levels: the need for a new classification scheme. Histochem Cell Biol.

[bib23] Gribble F.M., Reimann F. (2019). Function and mechanisms of enteroendocrine cells and gut hormones in metabolism. Nat Rev Endocrinol.

[bib24] Haber A.L., Biton M., Rogel N., Herbst R.H., Shekhar K., Smillie C., Burgin G., Delorey T.M., Howitt M.R., Katz Y. (2017). A single-cell survey of the small intestinal epithelium. Nature.

[bib25] Habib A.M., Richards P., Cairns L.S., Rogers G.J., Bannon C., Parker H.E., Morley T., Yeo G., Reimann F., Gribble F.M. (2012). Overlap of endocrine hormone expression in the mouse intestine revealed by transcriptional profiling and flow cytometry. Endocrinology.

[bib26] Hansen A., Reutter K., Zeiske E. (2002). Taste bud development in the zebrafish, Danio rerio. Dev Dynam.

[bib27] Hashiguchi Y., Furuta Y., Kawahara R., Nishida M. (2007). Diversification and adaptive evolution of putative sweet taste receptors in three spine stickleback. Gene.

[bib28] Hass N., Schwarzenbacher K., Breer H. (2010). T1R3 is expressed in brush cells and ghrelin-producing cells of murine stomach. Cell Tissue Res.

[bib29] Herness E., Johnson L.R. (2018). Physiology of the gastrointestinal tract.

[bib30] Herrera Moro Chao D., Argmann C., Van Eijk M., Boot R.G., Ottenhoff R., VanRoomen C., Foppen E., Siljee J.E., Unmehopa U.A., Kalsbeek A. (2016). Impact of obesity on taste receptor expression in extra-oral tissues: emphasis on hypothalamus and brainstem. Sci Rep.

[bib31] Hoang L., Angotzi A.R., Ebbesson L.O.E., Karlsen Ø., Rønnestad I. (2016). The ontogeny and brain distribution of the appetite regulators NPY, CART and pOX in larval Atlantic cod (*Gadus morhua* L.). PLoS One.

[bib32] Hoon M.A., Adler E., Lindemeier J., Battey F., Ryba N.J.P., Zuker C.S. (1999). Putative mammalian taste receptors: a class of taste-specific GPCRs with distinct topographic selectivity. Cell.

[bib33] Hughes S.G. (1991). Response of first-feeding spring chinook salmon to four potential chemical modifiers of feed intake. Progress Fish Cult.

[bib34] Hughes S.G. (1993). Single-feeding response of chinook salmon fry to potential feed intake modifiers. Progress Fish Cult.

[bib35] Jang H.J., Kokrashvili Z., Theodorakis M.J., Carlson O.D., Kim B.J., Zhou J., Kim H.H., Xu X., Chan S.L., Juhaszova M. (2007). Gut-expressed gustducin and taste receptors regulate secretion of glucagon-like peptide-1. Proc Natl Acad Sci USA.

[bib36] Kasumyan A.O. (2001). Functional development of chemosensory systems in the fish ontogeny. Russ J Dev Biol.

[bib37] Kasumyan A.O., Sidorov S.S. (2005). Taste preferences of the trout Salmo trutta from three geographically isolated populations. J Ichthyol.

[bib38] Kinnamon S.C. (2012). Taste receptor signalling from tongues to lungs. Acta Physiol.

[bib39] Kojima I., Nakagawa Y., Ohtsu Y., Medina A., Nagasawa M. (2014). Sweet taste-sensing receptors expressed in pancreatic β-cells: sweet molecules act as biased agonists. Endocrinol Metab.

[bib40] Kurokawa T., Suzuki T., Andoh T. (2000). Development of cholecystokinin and pancreatic polypeptide endocrine systems during the larval stage of Japanese flounder, *Paralichthys olivaceus*. Gen Comp Endocrinol.

[bib41] Lahnsteiner F. (2017). Digestive enzyme system of larvae of different freshwater teleosts and its differentiation during the initial phase of exogenous feeding. Czech J Anim Sci.

[bib42] Latorre R., Mazzoni M., De Giorgio R., Vallorani C., Bonaldo A., Gatta P.P., Corinaldesi R., Ruggeri E., Bernardini C., Chiocchetti R. (2013). Enteroendocrine profile of a-transducin immunoreactive cells in the gastrointestinal tract of the European sea bass (Dicentrarchuslabrax). Fish Physiol Biochem.

[bib43] Latorre R., Sternini C., De Giorgio R., Greenwood-Van Meerveld B. (2016). Enteroendocrine cells: a review of their role in brain-gut communication. Neuro Gastroenterol Motil.

[bib44] Lee R.J., Cohen N.A. (2015). Taste receptors in innate immunity. Cell Mol Life Sci.

[bib45] Li H.J., Ray S.K., Pan N., Haigh J., Fritzsch B., Leiter A.B. (2019). Intestinal Neurod1 expression impairs paneth cell differentiation and promotes enteroendocrine lineage specification. Sci Rep.

[bib46] Li H.J., Ray S.K., Singh N.K., Johnston B., Leiter A.B. (2011). Basic helix-loop-helix transcription factors and enteroendocrine cell differentiation. Diabetes Obes Metabol.

[bib47] Li X. (2009). T1R receptors mediate mammalian sweet and umami taste. Am J Clin Nutr.

[bib48] Li X., Staszewski L., Xu H., Durick K., Zoller M., Adler E. (2002). Human receptors for sweet and umami taste. Proc Natl Acad Sci USA.

[bib49] Lindemann B. (2001). Receptors and transduction in taste. Nature.

[bib50] Mafi A.H., Kim S.K., Keng C., Chou K.C., Güthrie B., Goddard W.A. (2021). Predicted structure of fully activated Tas1R3/1R3′ homodimer bound to g protein and natural sugars: structural insights into G protein activation by a class c sweet taste homodimer with natural sugars. J Am Chem Soc.

[bib51] Masubuchi Y., Nakagawa Y., Ma J., Sasaki T., Kitamura T., Yamamoto Y., Kurose H., Kojima I., Shibata H. (2013). A novel regulatory function of sweet taste-sensing receptor in adipogenic differentiation of 3T3-L1 cells. PLoS One.

[bib52] McCaughey S.A. (2021). Variation in the gene Tas1r3 reveals complex temporal properties of mouse brainstem taste responses to sweeteners. Am J Physiol Regul Integr Comp Physiol.

[bib53] Morais S. (2017). The physiology of taste in fish: potential implications for feeding stimulation and gut chemical sensing. Rev Fish Sci Aquac.

[bib54] Moyano F.J., Díaz M., Alarcon F.J., Sarasquete C. (1996). Characterization of digestive enzyme activity during larval development of gilthead seabream, *Sparus aurata* L. Fish Physiol Biochem.

[bib55] Muller P., Janovjak H., Miserez A., Dobbie Z. (2002). Processing of gene expression data generated by quantitative real-time RT-PCR. Biotechniques.

[bib56] Nelson G., Chandrashekar J., Hoon M.A., Feng L., Zhao G., Ryba N.J., Zuker C.S. (2002). An amino-acid taste receptor. Nature.

[bib57] Nelson G., Hoon M.A., Chandrashekar J., Zhang Y., Ryba N.J., Zuker C.S. (2001). Mammalian sweet taste receptors. Cell.

[bib58] Ohmoto M., Okada S., Nakamura S., Abe K., Matsumoto I. (2011). Mutually exclusive expression of Gαia and Gα14 reveals diversification of taste receptor cells in zebrafish. J Comp Neurol.

[bib59] Oike H., Nagai T., Furuyama A., Okada S., Aihara Y., Ishimaru Y., Marui T., Matsumoto I., Misaka T., Abe K. (2007). Characterization of ligands for fish taste receptors. J Neurosci.

[bib60] Oka Y., Korsching S.I. (2011). Shared and unique Gg alpha proteins in the zebrafish *versus* mammalian senses of taste and smell. Chem Senses.

[bib62] Otero-Rodiño C., Librán-Pérez M., Velasco C., López-Patiño M.A., Míguez J.M., Soengas J.L. (2015). Evidence for the presence of glucosensor mechanisms not dependent on glucokinase in hypothalamus and hindbrain of rainbow trout (*Oncorhynchus mykiss*). PLoS One.

[bib63] Palazzi R., Richard J., Bozzato G., Zanella L. (2006). Larval and juvenile rearing of common sole (*Solea solea* L.) in the Northern Adriatic (Italy). Aquaculture.

[bib64] Ping H.C., Feng K., Zhang G.R., Wei K.J., Zou G.W., Wang W.M. (2014). Ontogeny expression of ghrelin, neuropeptide Y and cholecystokinin in blunt snout bream, *Megalobrama amblycephala*. J Anim Physiol Anim Nutr.

[bib65] Polakof S., Soengas J.L. (2013). Evidence of sugar sensitive genes in the gut of a carnivorous fish species. Comp Biochem Physiol B Biochem Mol Biol.

[bib66] Raka F., Farr S., Kelly J., Stoianov A., Adeli K. (2019). Metabolic control via nutrient-sensing mechanisms: role of taste receptors and the gut-brain neuroendocrine axis. Am J Physiol Endocrinol Metab.

[bib67] Raybould H.E. (2010). Gut chemosensing: interactions between gut endocrine cells and visceral afferents. Auton Neurosci.

[bib68] Ren X., Zhou L., Terwilliger R., Newton S.S., De Araujo I.E. (2009). Sweet taste signaling functions as a hypothalamic glucose sensor. Front Integr Neurosci.

[bib69] Ribeiro L., Zambonino-Infante J.L., Cahu C., Dinis M.T. (1999). Development of digestive enzymes in larvae of Solea senegalensis, Kaup 1858. Aquaculture.

[bib70] Ronnestad I., Jordal A.E.O., Gomes A.S. (2016). Nutrient sensing in Atlantic salmon-exploring the sensory systems for amino acids in the gastrointestinal tract. Faseb J.

[bib71] Rønnestad I., Yúfera M., Ueberschär B., Ribeiro L., Sæle Ø., Boglione C. (2013). Feeding behavior and digestive physiology in larval fish: current knowledge, and gaps and bottlenecks in research. Rev Aquac.

[bib72] Roper S. (1989). The cell biology of vertebrate taste receptors. Annu Rev Neurosci.

[bib73] Sbarbati A., Osculati F. (2003). Solitary chemosensory cells in mammals?. Cells Tissues Organs.

[bib74] Sbarbati A., Osculati F. (2005). The taste cell-related diffuse chemosensory system. Prog Neurobiol.

[bib75] Simon P. (2003). Q-Gene: processing quantitative real-time RT–PCR data. Bioinformatics.

[bib76] Sjölund K., Sandén G., Håkanson R., Sundler F. (1983). Endocrine cells in human intestine: an immunocytochemical study. Gastroenterology.

[bib77] Sternini C. (2007). Taste receptors in the gastrointestinal tract. IV. Functional implications of bitter taste receptors in gastrointestinal chemosensing. Am J Physiol Gastrointest Liver Physiol.

[bib78] Sun X., Fu X., Du M., Zhu M. (2018). Ex vivo gut culture for studying differentiation and migration of small intestinal epithelial cells. Biol Open.

[bib79] Taniguchi K. (2004). Expression of the sweet receptor protein, T1R3, in the human liver and pancreas. J Vet Med Sci.

[bib80] Thisse C., Thisse B. (2008). High-resolution in situ hybridization to whole-mount zebrafish embryos. Nat Protoc.

[bib81] Triantafillou V., Workman A.D., Kohanski M.A., Cohen N.A. (2018). Taste receptor polymorphisms and immune response: a review of receptor genotypic-phenotypic variations and their relevance to chronic rhinosinusitis. Front Cell Infect Microbiol.

[bib82] Uhlen M., Fagerberg L., Hallstrom B.M., Lindskog C., Oksvold P., Mardinoglu A., Sivertsson A., Kampf C., Sjostedt E., Asplund A. (2015). Tissue-based map of the human proteome. Science.

[bib83] Varatharasan N., Croll R.P., Franz-Odendaal T. (2009). Taste bud development and patterning in sighted and blind morphs of *Astyanax mexicanus*. Dev Dynam.

[bib84] Wang C., Ma J.Z., Xu Q., Yin J., Li J., Wang L., Zhao Z.G., Luo L. (2016). The development of pharyngeal taste buds in *Hucho taimen* (Pallas, 1773) larvae. IJFS (Ital J Food Sci).

[bib85] Yarmolinsky D.A., Zuker C.S., Ryba N.J. (2009). Common sense about taste: from mammals to insects. Cell.

[bib86] Ye L., Mueller O., Bagwell J., Bagnat M., Liddle R.A., Rawls J.F. (2019). High fat diet induces microbiota-dependent silencing of enteroendocrine cells. Elife.

[bib87] Young R.L. (2011). Sensing via intestinal sweet taste pathways. Front Neurosci.

[bib88] Yuan X., Liang X.F., Cai W.J., He S., Guo W.J., Mai K.S. (2020). Expansion of sweet taste receptor genes in grass carp (*Ctenopharyn godonidellus*) coincided with vegetarian adaptation. BMC Evol Biol.

[bib89] Yúfera M., Fernández-Díaz C., Pascual E. (2005). Food microparticles for larval fish prepared by internal gelation. Aquaculture.

[bib90] Yúfera M., Darias M.J. (2007). The onset of exogenous feeding in marine fish larvae. Aquaculture.

[bib91] Zambonino-Infante J., Gisbert E., Sarasquete C., Navarro I., Gutíerrez J., Cahu C.L., Cyrino J., Bureau D., Kapoor B.G. (2008). Feeding and digestive functions of fishes.

[bib92] Zambonino-Infante J.L., Cahu C.L. (2001). Ontogeny of the gastrointestinal tract of marine fish larvae. Comp Biochem Physiol, C.

