# Supplementary material for: Exploring the potential for an evolutionarily conserved role of the taste 1 receptor gene family in gut sensing mechanisms of fish
Source: Anim Nutr. 2022 Aug 31;11:293–308. doi: 10.1016/j.aninu.2022.08.010 (PMC9563615; doi:10.1016/j.aninu.2022.08.010)
Supplement: Multimedia component 1 [file mmc1.docx]

Supplementary Table S1. Primer sequences

| **Gene** | **Accession number** | **Forward primer (5'→3')** | **Reverse primer (5'→3')** | **Applications** |
| --- | --- | --- | --- | --- |
| *ef2* | KY388506 | TGCACAAAAGTACCGTTGTGACCTG | AATGCGAACTTTCAGACCAGTGGAG | qPCR |
| *T1R1* | XP_030277517 | CTGATGCAAGCCATGAGATTTGCTG | CCAATCAAAGCCAATCCTCTCTGA | qPCR |
| *T1R2a* | XP_030278006 | AGGAGTGATTGTTGAGATCATTCTGC | ACTCAATGAGAGCTTCAGCAGTCAAT | qPCR |
| *T1R2b* | XP_030278002 | CACTTACATAAGCACTGAAACACGGACTG | CATCTATGTTAAGGAAAGAAACCCAGCG | qPCR |
| *T1R2d* | XM_030422143 | GCGCAGAAGATGTAATCGCAACAA | CCTCAGCTCTGCTAGAACCACATGTGGGTA | qPCR |
| *T1R2e* | XM_030422144 | GTACAGAAGCCATCCTCGCTGCGG | CTTCAATTTGGCTAGAACCATGTGTGGGTA | qPCR |
| *T1R2f* | XM_030422145 | CGGCAAAAGATGTCATTCTTGCAG | TTTCAGCTCTGCTAGAACCATGTATGGAAA | qPCR |
| *T1R3* | XP_030274769 | GGAGACATTATCCTCGGAGGGCTCT | TTCCGAGCTATAAGGACCGATCACGGCT | qPCR |
| *T1R1* | XP_030277517 | CTGATGCAAGCCATGAGATTTGCTG | GACCAGTCCTCTGACCCGATCCATACCTT | ISH-probe (710 bp) |
| *T1R2a* | XP_030278006 | AGATTGGTAAATGGTAACTACAGCAATG | ACTCAATGAGAGCTTCAGCAGTCAAT | ISH-probe (845 bp) |
| *T1R2b* | XP_030278002 | ACAACAATGAAGCACTGCCTGGCTT | CATCTATGTTAAGGAAAGAAACCCAGCG | ISH-probe (645 bp) |
| *T1R2d* | XM_030422143 | TCCATAGAGGTGATTTATCACATTGTGCAGC | CCTCAGCTCTGCTAGAACCACATGTGGGTA | ISH-probe (600 bp) |
| *T1R2e* | XM_030422144 | GTCATAGAGGTGATTGTTAGCATCATACAGC | CTTCAATTTGGCTAGAACCATGTGTGGGTA | ISH-probe (600 bp) |
| *T1R2f* | XM_030422145 | ATCATAGAAGTGATTGTTAGGATTCTGCAGC | TTTCAGCTCTGCTAGAACCATGTATGGAAA | ISH-probe (600 bp) |
| *T1R3* | XP_030274769 | CTGCCTCTCACCTGCATGTACTCA | TTACATGACTTTTGATGATTGCTCTGATTCTG | ISH-probe (588 bp) |
| *Giα1* | XP_030281956 | CATGGGGTGCACCCTGAGCA | TTAGAAGAGGCCGCAGTCCTTTAGGTTG | ISH-probe (1,065 bp) |
| *Giα2* | XP_030276216 | ATGGGTTCACGGTGAGCGCCGAGGATAA | TTAGAAGAGGCCGCAGTCCTTTAGGTTG | ISH-probe (1,068 bp) |
| *ghre* | MG570187 | AACACCTGTTTGCTGGTGTT | ATCTTCAGAGTTGTGGCGGTC | ISH-probe (310 bp) |
| *cck* | KP822925 | TGTCGTGCTGGCGGTCCTGTGTA | AAGTAGTCCCTGTCTGCTATCCG | ISH-probe (336 bp) |
| *pg* | XM_030429139 | GAAAAGCGCTCACTCTTTGGCTGGACTC | GGTCTTCAGCCAGGACACGAACT | ISH-probe (369 bp) |
| *pyy* | XM_030407807 | ATGGCTGTGATGCTAAAGCCATGGAC | CCACACGTAGGAGTCGTCATATCTTGATC | ISH-probe (291 bp) |
| *nd1* | XM_030429258 | ATGACGAAGAAGAGGAGGACGAAGAC | ATGAGAAGTTCCCGTTGATGCTCAGT | ISH-probe (654 bp) |
| *pomcβ* | HM584910 | ATGTGTCCTGTGTGGCTATTGGTGG | TCACTGCTGCTGCTGTCCGTCTTTGTT | ISH-probe (699 bp) |

Supplementary Table S2. Probe specificity

| Gene | *T1R1* | *T1R2a* | *T1R2b* | *T1R2d* | *T1R2e* | *T1R2f* | *T1R3* |
| --- | --- | --- | --- | --- | --- | --- | --- |
| *T1R3* | 55.52 | 44.63 | 45.89 | 48.59 | 47.98 | 47.35 | 42.59 |
| *T1R2a* | 50.39 | 41.48 | 41.56 | 42.01 | 41.50 | 41.62 | 42.77 |
| *T1R2b* | 52.55 | 79.18 | 79.97 | 83.31 | 81.36 | 82.64 | 41.96 |
| *T1R2d* | 50.00 | 77.48 | 79.47 | 83.31 | 83.76 | 87.67 | 43.26 |
| *T1R2e* | 50.40 | 78.90 | 82.89 | 83.76 | 83.76 | 83.85 | 43.18 |
| *T1R2f* | 50.64 | 80.65 | 89.91 | 83.85 | 83.84 | 83.84 | 42.74 |

Percentage (%) of nucleotide conservation of cRNA probe sequence targets for a given *sa*T1R versus homologous gene regions of *sa*T1R gene paralogs. *sa*T1R = seabream taste 1 receptor.
